# Application of NMR and Chemometrics for the Profiling and Classification of Ale and Lager American Craft Beer

**DOI:** 10.3390/foods10040807

**Published:** 2021-04-09

**Authors:** Morgan Vasas, Fenfen Tang, Emmanuel Hatzakis

**Affiliations:** 1 Department of Food Science and Technology, The Ohio State University, Columbus, OH 43210, USA; morganvasas@gmail.com (M.V.); tang.1263@buckeyemail.osu.edu (F.T.); 2 Foods for Health Discovery Theme, The Ohio State University, Columbus, OH 43210, USA

**Keywords:** craft beer, ale, lager, NMR, chemometrics

## Abstract

In this paper, Nuclear Magnetic Resonance spectroscopy (NMR)-based metabolomics were applied for the discrimination of ale and lager craft American beers. A modified pulse sequence that allows the efficient suppression of the water and ethanol peaks was used to achieve high-quality spectra with minimal sample preparation. The initial chemometrics analysis generated models of low predictive power, indicating the high variability in the groups. Due to this variability, we tested the effect of various data pretreatment and chemometrics approaches to improve the model’s performance. Spectral alignment was found to improve the classification significantly, while the type of normalization also played an important role. NMR combined with statistical and machine-learning techniques such as orthogonal projection to latent structures discriminant analysis (OPLS-DA) and random forest was able to discriminate between ale and lager beers, thus providing an important tool for the quality control and analysis of these products.

## 1. Introduction

Beer is a popular beverage throughout the world. It has a significant impact on both economy and human society. Beers exist in many varieties; however, they are commonly categorized into two discrete groups, i.e., top- and bottom-fermented beers, known as ales and lagers, respectively. This classification is drawn from the strain of yeast utilized in the fermentation process and its behavior within the fermentation tank. Top fermentation involves the yeast *Saccharomyces cerevisiae*; such beers are called “top fermenting” due to the propensity of this strain of yeast to float naturally to the top of the fermentation tun. Likewise, bottom fermentation, associated with the production of lager-type beers, involves the yeast *Saccharomyces pastorianus* that typically sinks to the bottom of the tank during fermentation [1].

Outside of fermentation, there are many additional process steps involved in brewing that determine the quality and properties of beer. The mashing step represents a fundamental part of the brewing process. This step generates fermentable sugars that can be broken down by yeast and develops many additional long-chain sugars and nonfermentable products, such as organic acids [2]. Within the mashing process, starches from a primary carbohydrate source, often a cereal crop such as barley or wheat, are broken down by amylolytic enzymes, chiefly, α- and β-amylase [3], through hydrolysis. Hence, the enzymatic process of hydrolysis is responsible for much of the character of beer.

In addition to the mainstream large-scale brewing industry, craft brewing production, characterized by its small scale and unique products, is constantly growing. The craft brewing industry in the United States has seen accelerated growth. According to the Brewer’s Association, US craft beer saw a volume growth of almost 4% in 2019 as compared to a declination of 2% in overall beer volume in the United States, which includes large-scale commercial breweries [4]. With the expansion of craft brewing, new approaches to the development of distinctive profiles and quality control in a beer product are being sought. Though not readily available to most craft breweries in-house due to cost, there is an interest in analytical assessments to enhance or validate methods of development [5] at craft scale.

Powerful analytical tools are required to understand the impact of various processes and formulations on the metabolite profile and, consequently, the quality and properties of different types of beer. Chemometrics analysis on data obtained by analytical platforms such as NMR, mass spectrometry (MS), and infrared spectroscopy (IR) is an emerging and promising tool for the quality control of food and beverage products, including beer [6,7]. IR and MS are mostly used; however, NMR has gained attention recently due to its many advantages, such as its non-destructive nature, high reproducibility, and unique structural analysis capabilities. There are many successful food-related applications of this approach, especially for detecting adulteration, determining the geographical origin, and assessing the effects of different processing parameters [8,9].

In many of those cases, there is a large difference in chemical composition among groups, and the factors determining the class membership are the main sources of variance. As a result, clustering and classification using chemometrics is often expected and easily achieved. Unfortunately, there are cases where the examined factor may not be the main or the only major source of variance, which can present a challenge to the analysis. Recently, ^1^H NMR combined with principal component analysis (PCA) and (O)PLS-DA was used for the discrimination of Brazilian lager beers [10,11], tracing the origin of Trappist beers [12], and for the discrimination between craft and industrial beers [13], showing the power of this approach for the quality control of beer. Since beer is a complex mixture consisting mainly of organic acids, alcohols, amino acids, and carbohydrates, it is an excellent system to test the potential and/or the limitations of analytical and chemometrics methods. Here, we hypothesized that the untargeted NMR profiling, which involves a holistic sample analysis, can be used for the classification of craft beers according to the type of fermentation, namely, top- (ale) and bottom- (lager) fermented beers. This task is challenged by the presence of several subgroups among beers produced by the two main brewing styles, as well as by additional factors of variance including different origins and primary carbohydrate sources. This task may be even more challenging for craft beers, for which diverse production practices and ingredients are used. 

The global NMR spectra of beer provide a wealth of information about the composition of each sample, including the metabolites associated with the differences in lager and ale [14]. In addition, more knowledge is required about the NMR data pretreatment and analysis to conduct a powerful statistical analysis. As such, the aim of this study was to investigate if these resultant characteristics prove sufficient for the discrimination of ale and lager craft beers and, as a result, have the potential to serve as indicators for the extent of fermentation and the generation of certain non-fermentable products. If successful, NMR analysis could be a useful method for advanced process development and quality control and may have the potential to be applied in lower magnetic fields, which fit better in food laboratories. This knowledge can help the brewing industry to better understand the influence of processing factors and different ingredients on beer composition and monitor brewing processes so that new processes may be developed or optimized. Further, we evaluated the potential of different approaches for NMR data treatment and analysis and assessed their effects on chemometric analysis.

## 2. Materials and Methods

### 2.1. Reagents and Beer Samples

D_2_O, trimethylsilylpropanoic acid d_4_ (TSP d_4_), K_2_HPO_4_, KH_2_PO_4_, sodium azide (NaN_3_), wheat starch, and alpha-amylase were purchased from Sigma-Aldrich (St. Louis, MO, USA). Amyloglucosidase was purchased from Diazyme Laboratories (Poway, CA, USA). Forty unique American craft beers were purchased from local stores in Columbus, Ohio. Further information about the samples can be found in Table S1. After initial run and spectral inspection, one outlier sample was removed due to a technical issue, leaving 19 lager samples and 20 ale samples. The mash sample was provided by North High Brewing.

### 2.2. Sample Preparation 

To prepare the samples, 25–30 mL of each sample was transferred to 45 mL Falcon tubes and agitated to degas. The degassing procedure consisted of 1 min of manual shaking and release of gasses, followed by 5 min of ultrasonication. Then, 570 µL of sample and 50 µL of phosphate buffer (100 mM K_2_HPO_4_/KH_2_PO_4_, pH 5.4) in D_2_O containing 10 mM of TSP d_4_ and 10 mM NaN_3_ were added into an NMR tube. Samples were stored in the freezer at −20 °C. 

### 2.3. Enzymatic Hydrolysis 

Partial hydrolysis of wheat starch was performed by dissolving 0.2 g of starch in 100 mL of phosphate-buffered saline (PBS) buffer, which was adjusted to a pH of 5.5 using 1 M hydrochloric acid. The solution was stirred until the starch was fully suspended in water, resulting in a cloudy solution. The solution was then heated to 68 °C. At this point, the solution appeared nearly transparent, indicating gelatinization. The solution was then placed in a 68 °C water bath, and 3 mL of alpha-amylase (0.69 U/mL) solution was added. The solution was allowed to incubate for 1 h to ensure sufficient alpha-amylase hydrolysis. An additional sample of starch was prepared that underwent partial alpha-amylase hydrolysis followed by a total hydrolysis using a solution of amyloglucosidase (Diazyme) added in excess and allowed to incubate for an additional 10 min. For each sample, 570 µL of the solution was transferred to a 5 mm NMR tube, and 30 µL of deuterated methanol (CD_3_OD) was added. Samples were stored in the freezer until analysis.

### 2.4. NMR Analysis

Instrumentation: NMR experiments were conducted on an 800 MHz Bruker NMR spectrometer equipped with a triple resonance inverse (TCI) helium-cooled 5 mm probe (Billerica, MA, USA) at 25 ± 0.1 °C. ^1^H NMR spectra for untargeted analysis were acquired using 4 dummy scans, 32 scans, a delay between pulses of 3 sec, and 64K data points using a modified pulse sequence that combines the first increment of NOESY sequence with presaturation (NOESYPR1D), with Water suppression Enhanced through T1 effects (WET) sequence. Then, 2D (heteronuclear single quantum coherence-total correlation spectroscopy) HSQC-TOCSY was run using the standard hsqcdietgpsisp.2 Bruker pulse sequence with 32 scans, 512 data points in F1, and a mixing time of 60 ms. Diffusion-ordered spectroscopy (DOSY) NMR experiments were run using the LED sequence with bipolar gradient pulses and presaturation (ledbpgppr2s) using 16 scans and 16 data points in F1 dimension. A δ of 1000 μs and a Δ of 60 ms were used, and pulse gradients were incremented from 2 to 95% of the maximum gradient strength in a linear ramp. The spectra were processed by the Topspin software package provided by Bruker Topspin.

### 2.5. Multivariate Data Analysis

For untargeted analysis, the spectral regions *δ* 0.50–9.50 were integrated into regions (bins) with equal width using the AMIX software package (V3.9, Bruker topspin, Billerica, MA, USA) and into bins of variable sizes using the R package mrbin v1.3.0 [15]. The different bin sizes were tested to evaluate their impact on the analysis. Multivariate statistical analysis (MVSA) was performed with R environment (RStudio, Version 4.0.2, Boston, MA, USA), SIMCA-P+ software (version 14.1, Umetrics, Sweden) and Metaboanalyst [16]. The performance of the OPLS-DA and random forests (RF) models was evaluated using various metrics such as R^2^, Q^2^, and out-of-bag (OOB) error, respectively. For RF tree construction, one-third of the instances were left out of the bootstrap sampling and were used as test data to measure the classification OOB. 

## 3. Results

### 3.1. NMR Analysis

Beer samples contain water and high amounts of ethanol that may distract any type of targeted or untargeted NMR analysis. The presence of the strong alcohol or water peaks in the spectrum may mask the effects of other metabolites with high relevance. While considered, solvent removal in a rotovap or a freeze dryer was not the preferred method for this study due to its removal of volatiles and potential for compound degradation, as well as making the analysis more laborious and time-consuming. In addition, it introduces one more factor of variance, which should ideally be avoided in an untargeted metabolomics analysis. For solvent suppression in metabolomics studies, the first increment of NOESY sequence with presaturation (NOESY1DPR) is generally used; however, in our opinion, this sequence is not appropriate for the NMR analysis of beer samples due to the presence of multiple solvent peaks. Therefore, a modified pulse sequence for multiple solvent suppression was successfully used for the simultaneous suppression of water and ethanol peaks without the obstruction of key metabolite resonances. This experiment incorporates the WET sequence into the NOESYPR1D for the suppression of ethanol peaks, while GARP4 is applied with reduced power for ^13^C decoupling during WET to allow the recording of high-resolution spectra. Figure S1 shows the comparison between spectra obtained with the standard NOESYPR1D experiment and the modified sequence and displays the better performance of the latter. In addition, the modified sequence performs better than WET because hydrogen chemical exchange between water and ethanol generates a broad water peak at *δ* 4.8, which reduces the efficiency of WET in suppressing the water signal.

**Figure 1:**
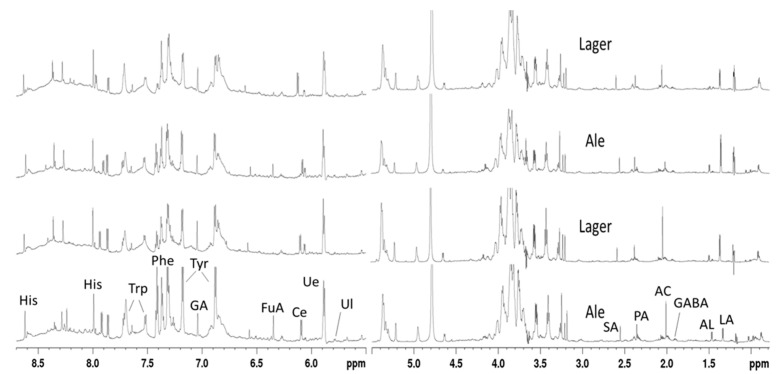
Representative 800 MHz ^1^H NMR spectra of ale and lager craft beers in phosphate buffer solution. LA, lactic acid; Al, alanine; GABA, gamma aminobutyric acid; AC acetate; PA, pyruvic acid; SA, succinic acid; Ul, uracil; Ue, uridine; Ce, cytidine; FuA, fumaric acid; Tyr, tyrosine; GA, gallic acid; Phe, phenylalanine; Trp, tryptophan; His, Histidine.

In a next step, we applied this method for the analysis of lager and ale craft beer samples. The method was proved to be effective even under high throughput conditions involving full automation. Representative normalized ^1^H NMR spectra of ale and lager craft beer are shown in Figure 1. Although a visual inspection indicates some differences in the levels of some metabolites among samples, it does not identify a certain pattern of the metabolite profile between groups. Additionally, extensive overlapping, especially in the carbohydrate region, makes definite peak visualization and identification difficult. As a result, differences between production styles can only be studied using chemometric analysis. 

NMR spectra of craft beer samples were similar to those recorded from other types of beer and characterized by several signals attributed to metabolites, such as succinic acid (*δ* 2.55), pyruvic acid (*δ* 2.36), acetic acid (*δ* 2.00), and lactic acid (*δ* 1.34). Other compounds such as alanine (*δ* 1.46), gamma aminobutyric acid (GABA) (*δ* 1.90, *δ* 2.39, *δ* 3.04), ethyl acetate (*δ* 1.22, *δ* 4.11), as well as isopentanol, isobutanol, and 1-propanol with overlapping signals at *δ* 0.86–0.91, dominate the high field region of the aliphatic area. The sugar region was dominated by the signals of fermentable sugars such as maltose and nonfermentable sugars like limit dextrins, while in the aromatic region, the signals of nucleosides such as uridine (*δ* 5.87, *δ* 7.86) and cytidine (*δ* 6.09, *δ* 7.91) and of amino acids such as histidine (*δ* 7.02), tyrosine (*δ* 7.17; *δ* 6.87), phenylalanine (*δ* 7.33, *δ* 7.38, *δ* 7.42), and tryptophan (*δ* 7.49) appeared. Other signals included those of trigonelline at *δ* 8.83, 9.11, and of formic acid at *δ* 8.42. These assignments are in agreement with previous studies [13,17], while 2D NMR was used for the confirmation of signals. As an example, Figure S2 shows the HSQC-TOCSY spectrum of a beer sample in the aliphatic region with some key assignments including the ^1^H and ^13^C resonances. 

**Figure 2:**
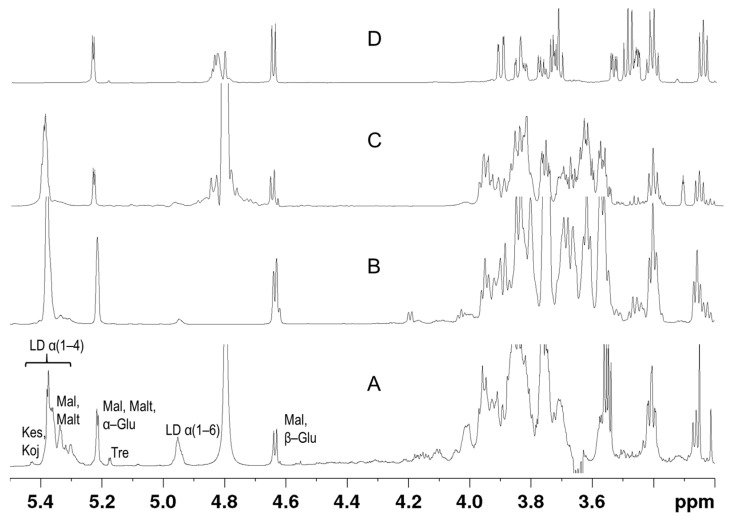
Shown are 800 MHz ^1^H NMR spectra of beer (**A**), mash (**B**), starch hydrolysis with α-amylase (**C**), and starch hydrolysis with amyloglucosidase (**D**).

The carbohydrate fraction is one of the most important components of beer and consists of carbohydrates of different sizes that mainly result from starch hydrolysis with α- and β-amylase. NMR can be used as an efficient tool for the rapid screening of carbohydrates in beer and thus for monitoring the degree of starch conversion to fermentable sugars and sugar fermentation under various conditions. Figure 2 compares the NMR spectra of beer with those obtained by the mashing process, starch hydrolysis with α-amylase, and total hydrolysis using α-amylase and amyl glucosidase, an enzyme that is exogenous to the mash and is used to increase the glucose content and consequently the availability of sugars for fermentation [18]. The carbohydrate profiles of beer and mash presented more similarities to those of partial hydrolysis with α-amylase, rather than to those of total hydrolysis which, as expected, produces mainly glucose. α-amylase mainly produces glucose, maltose, and maltotriose, while short peaks that belong to limit dextrins also appeared in the spectrum. Some of the beer carbohydrates can also be separated according to their diffusion coefficients, so the presence of carbohydrates with different degrees of polymerization can be studied by DOSY. Figure S3 shows the DOSY spectrum of a beer sample in the carbohydrate region. 

### 3.2. Chemometrics

#### 3.2.1. Initial Untargeted Analysis

Chemometric analysis was then applied using the ^1^H NMR data. The PCA plot obtained from the global NMR spectra using typical data treatment conditions is shown in Figure S4 and displays that there was no clear trend or clustering between sample groups. This observation indicates that the examined factor, i.e., the type of yeast/fermentation (ale vs. lager) was not the main source of variance and rendered the analysis more challenging. Other unavailable factors such as raw materials, brewing duration, and addition of adjuncts seemed to play a more important role compared to the type of fermentation. A test was conducted to determine if group discrimination was being hindered by varied configurations of sugars due to alpha-to-beta anomer interconversion in the spectral profile. To test this hypothesis, the samples were allowed to equilibrate at room temperature overnight. Subsequent MVSA showed no differences resulting from this test. In addition, the samples were analyzed based upon the packaging of each sample (bottle versus can); however, no discrimination was observed with NMR. PCA also revealed the existence of outliers, according to Hotelling’s T^2^ test. A common practice in studies involving chemometrics is the removal of outliers, often without any further consideration; however, this removal always needs to be done with caution. In this study, outliers were not excluded from the analysis, since inspection of the raw NMR data for those samples did not reveal any issues regarding spectral quality. Therefore, these samples may be indicative of future samples, and their removal would reduce the total variance. 

The use of a supervised method, namely, OPLS-DA, revealed a separation between classes with a *p* value for CV-ANOVA diagnostic of 0.008, indicating a significant model [19]. However, this model was not considered as totally reliable because the large difference between the values of cross validation metrics R^2^ (0.88) and Q^2^ (0.53) indicated some overfitting, since the model outperforms significantly for training data as compared to testing data. To examine the effect of binning on the model’s performance, different bin sizes were tested, and the results are shown in Table S2. A bin size of 0.05 ppm led to the weakest model, indicating that smaller bins can separate important signals that have similar chemical shifts. However, bin sizes smaller than 0.01 ppm were not found to significantly improve the model in terms of R^2^, Q^2^, and analysis of variance of the cross-validated residuals (CV-ANOVA) metrics, while the 0.005 ppm-based model was even weaker than the 0.01 ppm model. In addition, despite the differences in validation metrics, all models were considered of low to moderate performance. Also, a bin size of 0.001 ppm should be used with caution and only on data acquired in fields of 800 MHz or higher, because it generates a large number of bins which can cause problems in the analysis, such as increased computing time and challenges with multiple testing comparisons. 

Metabolites selected based on biological and/or molecular structure criteria can be used for classification purposes. For example, carbohydrates and phenolics have been used previously for beer classification [13,20,21,22]. Here, a small improvement was found when using the carbohydrate region of the spectrum (5.5–3 ppm) with an OPLS-DA model R^2^ = 0.85 and Q^2^ = 0.58. Also, selection of only the signals that appeared between 10.5 and 5.5 ppm and belonged mainly to polyphenols, aromatic amino acids, and aldehydes generated an OPLS-DA model with R^2^ = 0.99 and Q^2^ = 0.60. In addition to using structural criteria, we tested the performance of the models by selecting variables based on the *p* values obtained by the *t*-test. Table S3 shows the *p* values obtained with a bin size of 0.01 ppm. Removal of only 5% of NMR bins with the highest *p* values did not improve the performance of the model, with R^2^ and Q^2^ values that remained the same. When using only the 25% of variables having the lowest *p* values, there was an improvement in Q^2^ and in the R^2^/Q^2^ ratio. More specifically, the model had an R^2^ of 0.95 and a Q^2^ of 0.68, when UV scaling was used. When using pareto scaling, R^2^ and Q^2^ became 0.89 and 0.69, respectively. There was also an improvement in the group separation in the PCA (Figure S5); however, this analysis was not considered fully untargeted because only 25% of the total NMR bins was used. In our opinion, metabolite selection using either structural or statistical criteria should be used with caution when combined with chemometrics to conduct an untargeted analysis. 

#### 3.2.2. Model Improvement and Biomarker Selection

One of the main factors that determines the success of an untargeted NMR-based metabolomics approach involving spectral comparison is signal alignment. It is one of the basic steps before applying chemometrics to NMR data as it generally improves the quality and reliability of the analysis, while signal misalignment prevents the rigorous application of statistics. A common practice to ensure signal alignment is the addition of a buffer to correct for signal variations due to pH differences, as we also did in this study, followed by spectral chemical shift calibration using a standard compound. However, in certain cases, the position of the peak can contain valuable information about a system [23], while a buffer may affect the dynamic equilibrium between compounds. Therefore, we re-analyzed the samples without adding the buffer to test if the correction on chemical shift alignment had a negative impact on the statistical analysis. The results did not show any improved clustering in PCA, while the OPLS-DA model was even weaker (R^2^ = 0.72, Q^2^ = 0.30, CV-ANOVA *p* = 0.06).

**Figure 3:**
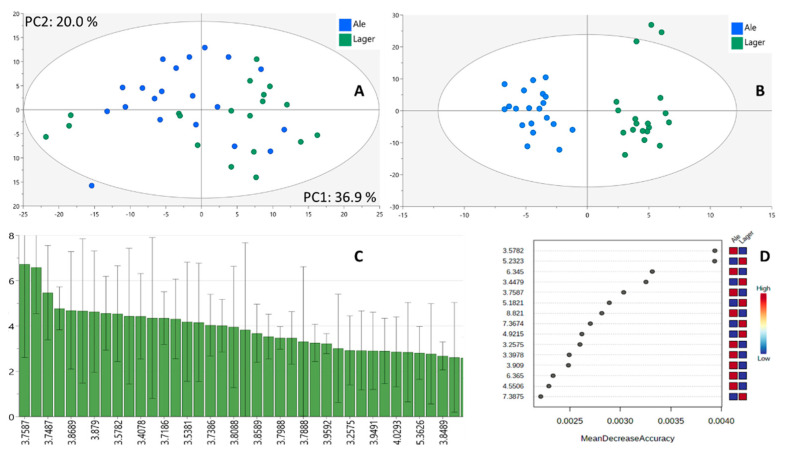
Principal component analysis (PCA) (**A**) and OPLS-DA scores plots (**B**) of ale and lager beers from 1D ^1^H NMR aligned using ICOSHIFT. Variable importance in projection (VIP) plot obtained by the OPLS-DA model showing the most significant variables for the classification (**C**) and variable importance plot obtained from random forest (**D**).

Since pH effects are not the only source of chemical shift variance in complex matrices such as beer, the addition of buffer does not fully correct for all signal misalignments, especially when smaller bin sizes are used. Thus, we repeated the analysis by correcting signal drifting using interval correlation optimized shifting (ICOSHIFT). ICOSHIFT is an algorithm first introduced by Sarovani et al. [23] and performs signal alignment in a rapid and user-friendly manner. Using ICOSHIFT, user-defined intervals were established around predominant peaks and peak clusters, and correlation shifting was performed algorithmically on each interval. Although only a slightly better clustering was observed in the PCA plot compared to non-aligned data, a significant improvement of the OPLS-DA model was found, as shown in Figure 3A,B. For the 0.01 ppm bin size an R^2^ of 0.92 and a Q^2^ of 0.69 were obtained, in contrast to 0.88 and 0.53 for the non-aligned data, while CV-ANOVA gave a *p* value of 6.63313 × 10^5^, showing a statistically significant separation between the groups. In addition, permutation tests indicated that there was no overfitting, as the R^2^ and Q^2^ values for permuted models were 0.67 and −0.79, respectively. Nevertheless, as shown in Figure 3A, both categories, namely, ales and lagers, displayed high dispersion in the PCA plot, indicating the significant variance in their chemical profiles. The value of signal alignment using ICOSHIFT should be evaluated case by case. Here, it was found to improve the classification; however, important information about the system can be lost because of peak alignment, as reported previously [23]. 

In the next step, we evaluated the impact of the sample normalization method on the OPLS-DA model’s performance. For the data of a bin size of 0.01 ppm where ICOSHIFT-based alignment was applied, probabilistic quotient normalization (PQN) was used and generated an OPLS-DA model with R^2^ = 0.96, Q^2^ = 0.70, and CV-ANOVA *p* value of 0.001. This model is of similar performance with the one described previously and obtained by normalization to total intensity (R^2^ = 0.92, Q^2^ = 0.69, CV-ANOVA *p* value = 6.63313 × 10^5^). It is important to note that, in this study, the same, exact amount of beer was used for all samples. In cases where the beer amount is not the same in the samples, a different normalization method may have a different impact on the model.

An alternative approach to correct for NMR signal drifting is to use variable bin sizes that allow the specific summation of only the peaks with variable chemical shifts. This pretreatment was conducted using the R package mrbin v1.3.0. Data were normalized using different methods, and the results are shown in Table 1. As shown, using this signal drifting correction methodology normalization using total intensity did not generate a strong OPLS-DA model, indicating that certain bins do not have an equal importance among samples. PQN generated a better model, while no normalized data that were subjected only to log transformation generated the best model using this approach. 

A combination of methods was used to find the most important biomarkers for ale vs. lager classification. The ICOSHIFT aligned data with a bin size of 0.01 ppm were used, as this generated OPLS-DA models with higher performance while also allowing for an accurate assignment of bins to certain metabolites. Figure 3C shows the variable importance in projection (VIP) plot of the most significant bins, and Table S4 shows the chemical shifts of the bins having a VIP value > 1. The most important bins presented chemical shifts of 3.2–4.0 ppm, 4.63–4.64, and 5.21–5.23, which belong to maltose and malto-oligosaccharides, as well as chemical shifts corresponding to α-(1–4)-limit dextrins, of 5.32–5.39 ppm. In addition to OPLS-DA, we also tested the ability of RF [24], another machine-learning method that is usually applied in a supervised model, for the classification of ale and lager craft beer samples. For the ICOSHIFT-aligned data with a bin size of 0.01 ppm, mean centered, and normalized to total intensity, the RF model gave an OOB error of 0.21 when using 1000 trees and 250 predictors (Table S5). The variable-importance plot obtained from RF using ICOSHIFT-aligned data with a bin size of 0.01 ppm is shown in Figure 3D. Using RF, a bin size of 0.001 ppm generated an OOB of 0.15, in contrast to OPLS-DA where the decrease in bin size did not improve the model’s performance.

Although similarities in predictors were found for both models, there were also differences between RF and OPLS-DA. For example, fumaric acid (*δ* 6.34) and trigonelline (*δ* 8.82), which are associated with ales and appeared as significant biomarkers from RF, were only considered as low-importance markers by OPLS-DA. Also, trehalose at *δ* 5.18, which was found to be strongly associated with lager, was identified as a medium importance metabolite by OPLS-DA analysis, with a VIP value of 1.27. A univariate analysis, namely *t*-test, was used for class comparison, using FDR correction for multiple testing comparison (Figure S6), and showed similar biomarkers as significant, but again there were differences compared to OPLS-DA and RF. For example, bins at *δ* 3.75 appeared as significant using *t*-test, OPLS-DA, and RF, but the bin at *δ* 2.38 (GABA) appearing as significant in the *t*-test, had a medium VIP value (1.38) and did not appear as an important marker in RF. 

In contrast to the common practices, the combination of more than one method for class comparison and for building prediction models may reveal additional information, so more than one statistical analysis method should be used. A limitation of the present study was the relatively small number of samples we used, which however, was adequate to show the potential of NMR for this analysis. Based on the findings of this research, additional studies using larger sample sizes from different origins and different processing practices are needed to build prediction and class comparison tools that can be used by the brewing industry.

## 4. Conclusions

NMR is an effective tool for the analysis of beer. A modified pulse sequence was successfully applied for the simultaneous suppression of water and ethanol peaks in a high-throughput manner. Chemometric analysis showed that there is a difference between the metabolite profile of craft ales and lagers, which allows for their classification. Although in the literature OPLS-DA models with Q^2^ values higher than 0.80 are often presented, that was not the case in this study, indicating that the overall classification based on yeast variety was not the main factor of variance. Different pretreatment approaches such as normalization and spectral alignment play an important role on a model’s performance, and they should be considered by researchers, especially when dealing with challenging systems such as the one described in this study. Although this case was challenging and the initial model was not ideal, by utilizing different data pretreatment approaches, we were able to produce a better model. 

## Figures and Tables

**Table 1:** Effect of normalization method on OPLS-DA model performance. PQN, probabilistic quotient normalization.

Normalization Method	R^2^/Q^2^ Values	CV-ANOVA (*p* Value)
Total intensity	0.87/0.51	0.013
Total intensity/Log transformation	0.94/0.45	0.011
PQN	0.90/0.64	0.002
PQN/Log transformation	0.99/0.63	0.052
No normalization	0.87/0.33	0.108
Log transformation	0.99/0.72	0.006

## Data Availability

The data presented in this study are available in the article and supplementary material.

## Supplementary Materials

The following are available online at https://www.mdpi.com/article/10.3390/foods10040807/s1, Figure S1: Comparison between spectra obtained with the standard NOESY1DPR experiment and the modified sequence used in this study; Figure S2: Signal assignment on a HSQC-TOCSY spectrum of beer; Figure S3: DOSY spectrum of a beer sample in the carbohydrate region; Figure S4: PCA scores plot of ale and lager beers analyzed by 1D 1H NMR in a phosphate buffer solution; Figure S5: PCA plot of ale and lager samples when using only the 25% of variables having the lowest *p* values as determined in a *t*-test; Figure S6: Biomarkers with a *p* value < 0.05 as obtained by a *t*-test using 1D 1H NMR data aligned with ICOSHIFT; Table S1: Beer samples used in this study; Table S2: Effect of binning size on OPLS-DA model performance; Table S3: *p* values obtained by a *t*-test when using a bin size of 0.01 ppm; Table S4: Bins with a VIP value > 1; Table S5: Confusion matrix obtained from random forest using ICOSHIFT aligned data with a bin size of 0.01 ppm.
