# Supplementary material for: Application of NMR and Chemometrics for the Profiling and Classification of Ale and Lager American Craft Beer
Source: Foods. 2021 Apr 9;10(4):807. doi: 10.3390/foods10040807 (PMC8069586; doi:10.3390/foods10040807)
Supplement: Supplementary file 1 [file foods-10-00807-s001.pdf]

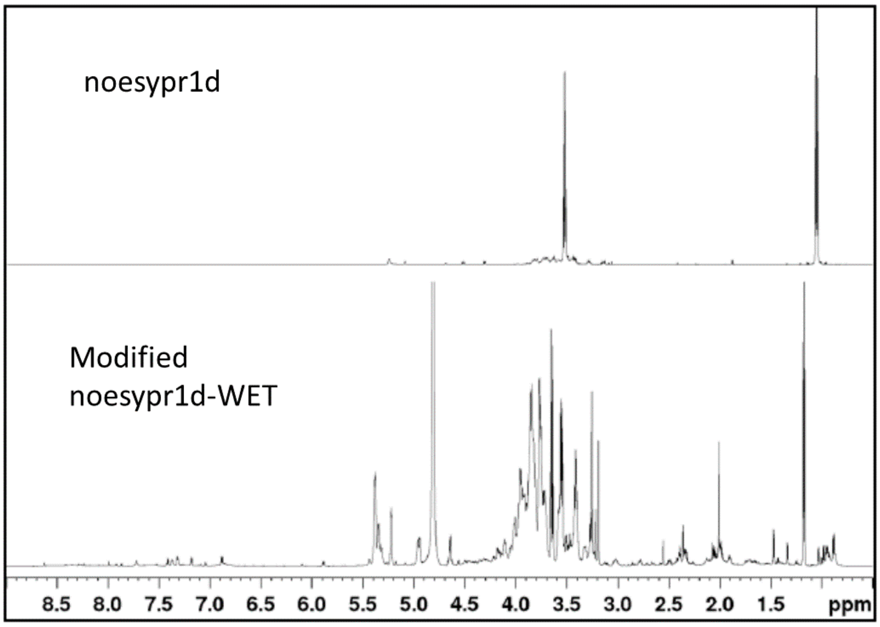

Figure S1. Comparison between spectra obtained with the standard NOESY1DPR experiment and the modified sequence used in this study.

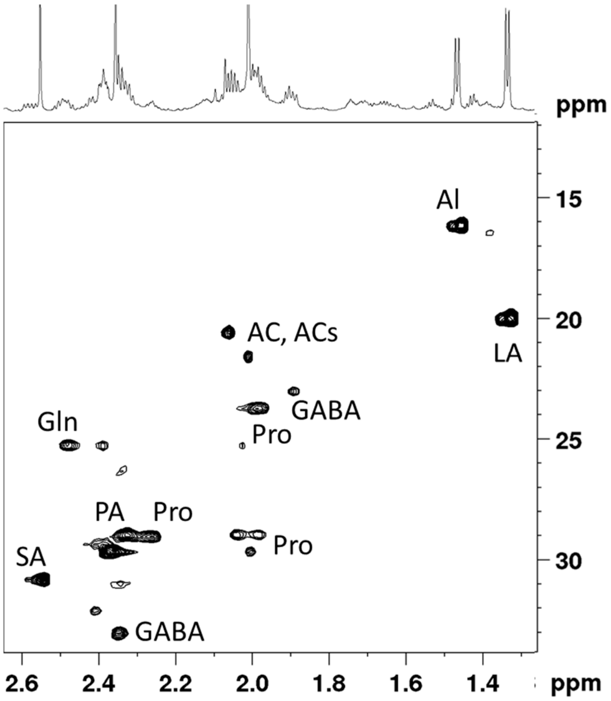

Figure S2. Signal assignment on a HSQC-TOCSY spectrum of beer; LA, lactic acid; Al, alanine; GABA, gamma aminobutyric acid; Pro, proline; AC/ACs, acetate(s); PA, pyruvic acid; Gln, glutamine; SA, succinic acid.

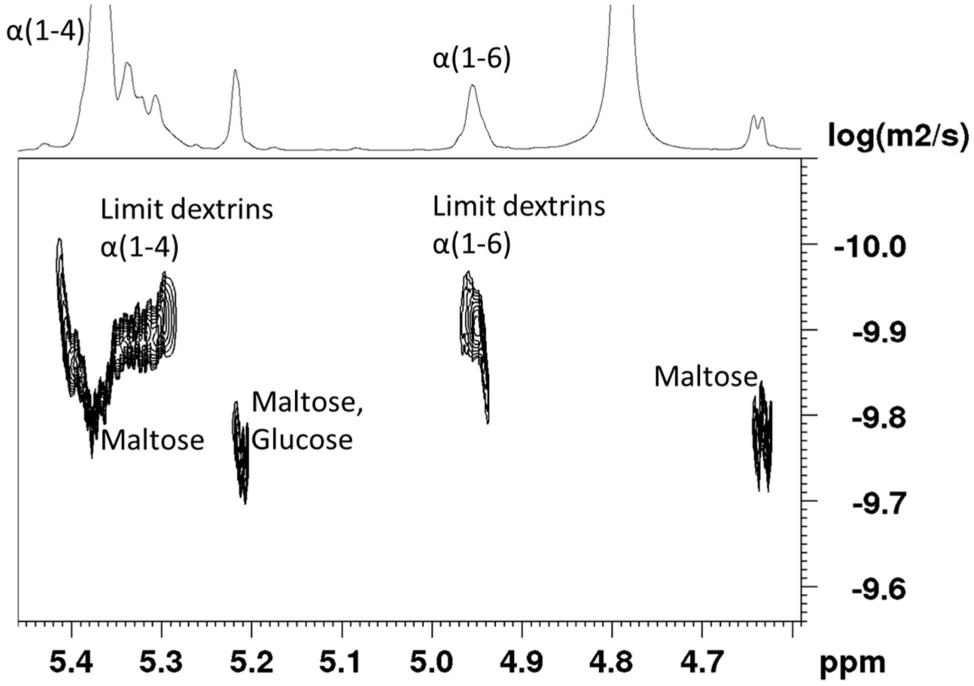

Figure S3. DOSY spectrum of a beer sample in the carbohydrate region.

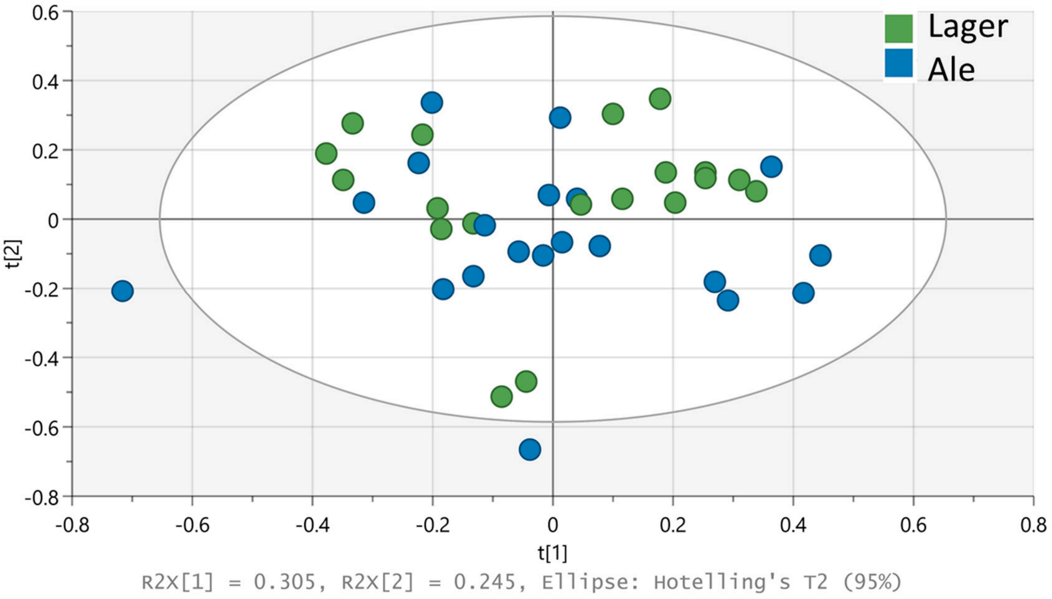

Figure S4. PCA scores plot of ale and lager beers analyzed by 1D ^{1}H NMR in a phosphate buffer solution. Bin size 0.01 ppm; normalization to total intensity.

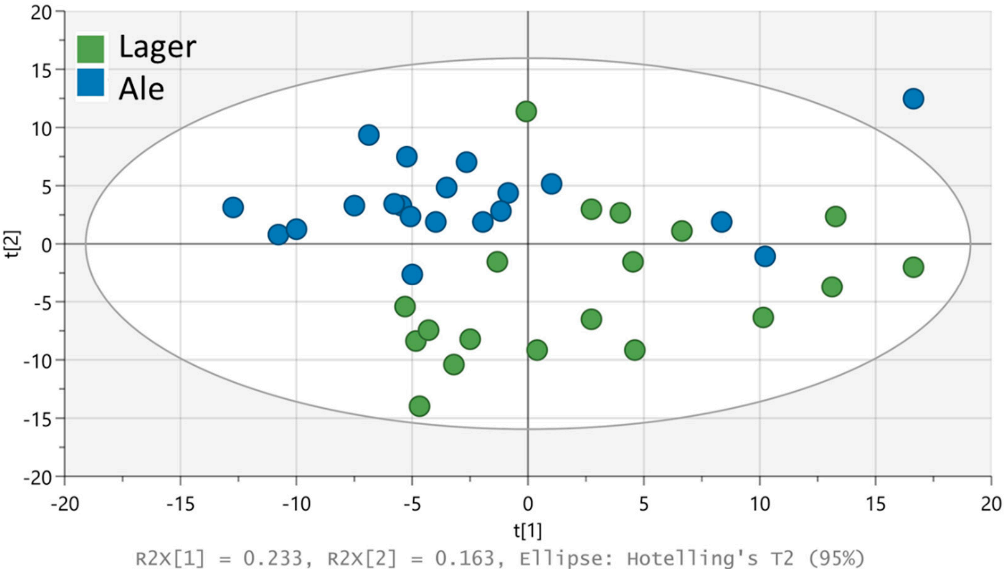

Figure S5. PCA plot of ale and lager samples when using only the 25% of variables having the lowest p values as determined in a t-test.

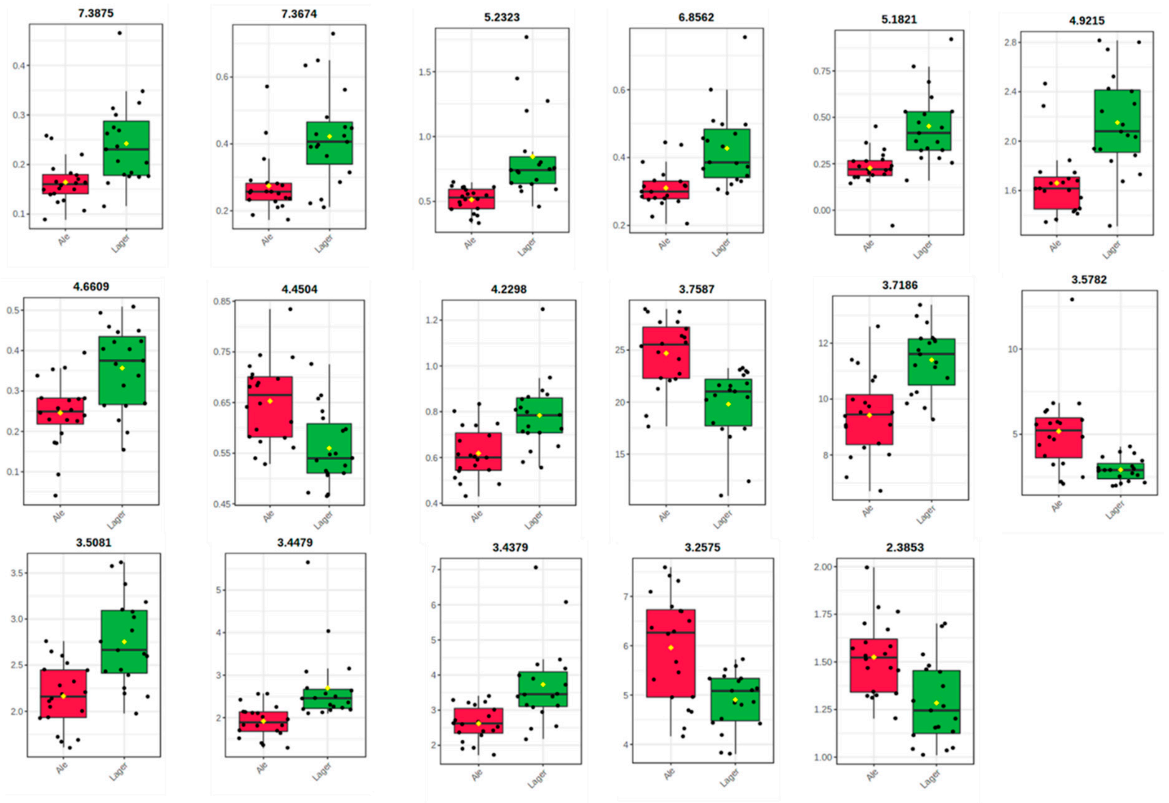

Figure S6. Biomarkers with a p value < 0.05 as obtained by a t-test using 1D 1H NMR data aligned with ICOSHIFT.

Table S1. Beer samples used in this study.

Region	Type	Special Variety	Alc %	Container
Athens, Ohio, USA	Ale	Amber ale	4.5	Can
Boston, MA, USA	Lager	Boston Lager	5	Bottle
Chicago, Illinois, USA	Ale	IPA	6.7	Can
Chicago, Illinois, USA	Lager	Barrel Aged Blonde Doppelbock	9.2	Bottle
Chico, California, USA	Lager	Czech Pilsner	5	Bottle
Cincinnati, Ohio, USA	Lager	Adjunct Lager	4.8	Can
Cincinnati, Ohio, USA	Ale	Kölsch-style	4.7	Can
Cincinnati, Ohio, USA	Ale	Pale Session Ale	4.5	Can
Cincinnati, Ohio, USA	Ale	IPA	7.2	Can
Cleveland, Ohio, USA	Lager	Dort	5.8	Bottle
Cleveland, Ohio, USA	Ale	Porter	6	Bottle
Cleveland, Ohio, USA	Ale	Imperial IPA	9.1	Bottle
Cleveland, Ohio, USA	Lager	Pale Lager	4.3	Can
Cleveland, Ohio, USA	Lager	Pilsner	5.5	Bottle
Columbus, Ohio, USA	Ale	American IPA	6	Bottle
Columbus, Ohio, USA	Ale	Porter	5.5	Can
Columbus, Ohio, USA	Ale	Pale Ale	6	Can
Columbus, Ohio, USA	Ale	Pale ale	5.5	Can
Columbus, Ohio, USA	Ale	Amber Ale/Red Ale	6.2	Can
Columbus, Ohio, USA	Lager	German Style Lager	5.3	Bottle
Columbus, Ohio, USA	Lager	Pilsner	5.5	Bottle
Comstock, Michigan, USA	Ale	IPA/High hop	7	Bottle
Dayton, Ohio, USA	Lager	Hoppy German Pilsner	5.4	Can
Dayton, Ohio, USA	Lager	Doppelbock	9.1	Can
Dayton, Ohio, USA	Lager	N/A	4	Can
Downington, Pennsylvania, USA	Lager	German Pilsner	5.3	Bottle
Fort Bragg, California, USA	Lager	Pilsner-style	4.7	Bottle
Grand Rapids, Michigan, USA	Ale	IPA	7	Can
Grand Rapids, Michigan, USA	Lager	American Adjunct style Lager	4.4	Can
Heath, Ohio, USA	Ale	Amber ale	5.2	Bottle
Holland, Michigan, USA	Ale	Brown Ale	6.5	Bottle
Holland, Michigan, USA	Ale	American/Midwest IPA	7	Bottle

Table S2. Effect of binning size on OPLS-DA model performance.

Bin size	R2/Q2 values	CV-ANOVA (p value)
0.05	0.83/0.46	0.036
0.01	0.88/0.53	0.008
0.005	0.89/0.51	0.01
0.001	0.99/0.56	0.04

Table S3. p values obtained by a t-test when using a bin size of 0.01 ppm.

ID (ppm)	p.value	FDR
4.655	0.00015	0.082784
4.665	0.000221	0.082784
1.205	0.000256	0.082784
3.465	0.000681	0.11746
6.855	0.000689	0.11746
6.345	0.000726	0.11746
3.985	0.000951	0.12449
0.845	0.001032	0.12449
3.455	0.001176	0.12449
5.235	0.00141	0.12449
4.935	0.001493	0.12449
0.835	0.001642	0.12449
4.635	0.001667	0.12449
3.895	0.003542	0.23294
1.535	0.003643	0.23294
8.405	0.003838	0.23294
3.915	0.0045	0.25705
0.855	0.00525	0.26821
6.845	0.005284	0.26821
5.225	0.005524	0.26821
4.625	0.0066	0.29287
6.365	0.006636	0.29287
2.955	0.007261	0.29582
6.375	0.008708	0.29582
2.165	0.009134	0.29582
3.265	0.009208	0.29582
7.385	0.009272	0.29582
2.935	0.009361	0.29582
6.505	0.00953	0.29582

6.435	0.010251	0.29582
3.425	0.010369	0.29582
3.475	0.010629	0.29582
8.325	0.011448	0.29582
6.905	0.011491	0.29582
0.825	0.011662	0.29582
7.645	0.013108	0.29582
0.795	0.013147	0.29582
9.105	0.013406	0.29582
7.625	0.013475	0.29582
0.815	0.013627	0.29582
2.155	0.013769	0.29582
3.385	0.013997	0.29582
0.805	0.014033	0.29582
4.555	0.014627	0.29582
3.565	0.014643	0.29582
6.355	0.014805	0.29582
8.435	0.015365	0.29582
8.365	0.015605	0.29582
3.755	0.016354	0.29582
2.525	0.016354	0.29582
6.395	0.016462	0.29582
4.905	0.016536	0.29582
6.415	0.017131	0.29582
1.805	0.017195	0.29582
5.085	0.017899	0.29582
5.295	0.018036	0.29582
1.525	0.018483	0.29582
0.905	0.018756	0.29582
3.395	0.018892	0.29582
5.395	0.018951	0.29582
1.425	0.019183	0.29582
3.445	0.019183	0.29582
2.695	0.019193	0.29582
3.405	0.02002	0.29723
8.815	0.020085	0.29723
5.215	0.020203	0.29723
8.335	0.022315	0.31479
6.515	0.022425	0.31479
3.515	0.023378	0.31479
8.825	0.023707	0.31479

8.085	0.023798	0.31479
0.865	0.02425	0.31479
8.135	0.024483	0.31479
6.425	0.024494	0.31479
5.705	0.024728	0.31479
8.395	0.024842	0.31479
6.405	0.024963	0.31479
5.435	0.026155	0.32559
2.255	0.02704	0.32966
4.445	0.02716	0.32966
2.175	0.027571	0.33051
7.035	0.027918	0.33059
7.665	0.02856	0.33412
6.545	0.029327	0.33901
3.255	0.029974	0.3424
2.815	0.031617	0.35591
3.495	0.031889	0.35591
5.275	0.032381	0.35694
8.355	0.032857	0.35694
7.695	0.033084	0.35694
3.825	0.03483	0.37165
0.785	0.037046	0.38777
4.615	0.03726	0.38777
7.635	0.037539	0.38777
6.915	0.038428	0.39277
8.375	0.039398	0.3957
6.335	0.03989	0.3957
3.575	0.040479	0.3957
3.505	0.040895	0.3957
3.745	0.041292	0.3957
3.485	0.041304	0.3957
7.655	0.041567	0.3957
5.105	0.042101	0.3969
2.925	0.043416	0.4042
4.225	0.044022	0.4042
3.725	0.044192	0.4042
4.675	0.044905	0.4042
4.545	0.044957	0.4042
1.815	0.046068	0.40987
6.445	0.046432	0.40987
8.445	0.047382	0.41449

0.775	0.048262	0.41497
8.195	0.048304	0.41497
8.385	0.048719	0.41497
5.405	0.050865	0.4268
4.925	0.051023	0.4268
3.905	0.051426	0.4268
8.315	0.05264	0.42781
5.675	0.053001	0.42781
4.455	0.053564	0.42781
2.465	0.053696	0.42781
2.215	0.053751	0.42781
7.615	0.055581	0.43715
3.555	0.056769	0.43715
8.125	0.056827	0.43715
4.215	0.056918	0.43715
5.285	0.057176	0.43715
8.835	0.058265	0.442
3.375	0.059382	0.44622
10.195	0.059742	0.44622
8.425	0.060846	0.45101
2.245	0.067128	0.49146
1.795	0.067316	0.49146
3.675	0.067981	0.49261
6.385	0.069029	0.49303
7.335	0.069055	0.49303
5.425	0.071321	0.49879
2.265	0.071438	0.49879
9.295	0.071507	0.49879
9.345	0.072346	0.49879
5.325	0.07243	0.49879
8.265	0.072993	0.49913
3.345	0.074469	0.50566
4.705	0.076605	0.51655
8.455	0.077484	0.51888
3.435	0.079306	0.52744
1.215	0.08093	0.53189
5.875	0.081229	0.53189
8.345	0.081964	0.53189
5.665	0.082166	0.53189
9.335	0.08304	0.53398
3.545	0.084008	0.53666

4.305	0.085012	0.53952
9.415	0.088062	0.54974
5.265	0.088195	0.54974
6.715	0.088322	0.54974
2.205	0.089518	0.55364
9.495	0.090404	0.55558
6.995	0.093483	0.56937
3.525	0.09382	0.56937
0.875	0.095505	0.576
2.765	0.0981	0.58799
6.065	0.10008	0.58977
8.095	0.10012	0.58977
2.475	0.1007	0.58977
9.485	0.10099	0.58977
7.225	0.10143	0.58977
5.205	0.10426	0.59758
0.915	0.10529	0.59758
5.965	0.10569	0.59758
3.715	0.10608	0.59758
7.275	0.10636	0.59758
7.375	0.10743	0.59758
6.705	0.10752	0.59758
7.085	0.10814	0.59758
2.705	0.10832	0.59758
2.865	0.10914	0.5987
0.765	0.11051	0.60285
4.435	0.11184	0.60533
10.215	0.11254	0.60533
8.555	0.11328	0.60533
2.655	0.11346	0.60533
2.885	0.11471	0.60866
7.065	0.11597	0.612
6.085	0.1177	0.61591
8.115	0.11829	0.61591
7.025	0.11885	0.61591
6.495	0.11925	0.61591
1.545	0.12116	0.62184
5.065	0.12468	0.62184
9.275	0.12522	0.62184
9.405	0.1254	0.62184
7.285	0.12653	0.62184

5.685	0.12713	0.62184
9.425	0.12773	0.62184
9.305	0.12805	0.62184
8.165	0.12806	0.62184
9.665	0.12955	0.62184
8.105	0.1301	0.62184
7.775	0.13023	0.62184
9.475	0.13059	0.62184
6.265	0.13129	0.62184
7.015	0.13153	0.62184
7.765	0.13174	0.62184
2.145	0.13175	0.62184
6.525	0.13217	0.62184
8.045	0.13257	0.62184
9.325	0.13396	0.62345
8.485	0.13466	0.62345
2.275	0.13528	0.62345
6.315	0.13548	0.62345
3.355	0.13981	0.64035
9.285	0.14157	0.64271
3.975	0.14188	0.64271
4.685	0.14231	0.64271
4.185	0.14315	0.64353
5.075	0.14604	0.64699
4.525	0.14634	0.64699
5.545	0.14656	0.64699
1.115	0.14686	0.64699
2.805	0.1488	0.64699
6.255	0.1492	0.64699
1.735	0.15016	0.64699
7.675	0.15046	0.64699
1.245	0.15087	0.64699
2.945	0.15111	0.64699
4.085	0.15125	0.64699
8.495	0.15484	0.65464
3.045	0.15524	0.65464
4.605	0.15527	0.65464
1.085	0.15574	0.65464
5.185	0.15712	0.65538
9.505	0.15848	0.65538
2.515	0.15914	0.65538

1.395	0.15918	0.65538
9.925	0.15929	0.65538
8.415	0.16033	0.65686
7.005	0.1612	0.65765
9.315	0.1668	0.66947
6.555	0.16779	0.66947
7.215	0.16831	0.66947
5.145	0.16864	0.66947
9.555	0.16886	0.66947
4.235	0.16923	0.66947
9.185	0.16938	0.66947
1.745	0.17058	0.66947
4.135	0.17271	0.66947
10.175	0.17325	0.66947
8.475	0.17327	0.66947
2.095	0.17381	0.66947
7.205	0.17433	0.66947
8.145	0.17483	0.66947
8.185	0.17531	0.66947
2.545	0.17554	0.66947
1.385	0.17649	0.66947
8.465	0.1765	0.66947
9.115	0.17761	0.67105
4.345	0.17965	0.67614
4.915	0.1808	0.67784
5.655	0.18469	0.68177
1.825	0.18545	0.68177
9.595	0.18552	0.68177
9.625	0.18561	0.68177
7.195	0.18709	0.68177
8.235	0.18726	0.68177
3.815	0.18741	0.68177
9.365	0.18747	0.68177
7.685	0.18901	0.68477
1.105	0.1897	0.68477
8.215	0.19077	0.68497
1.435	0.19117	0.68497
3.845	0.19278	0.68821
10.185	0.19458	0.68984
6.325	0.19466	0.68984
3.775	0.19651	0.69024

5.135	0.19711	0.69024
9.135	0.19927	0.69024
6.245	0.19931	0.69024
9.175	0.19933	0.69024
9.025	0.20037	0.69024
5.605	0.2008	0.69024
8.155	0.20187	0.69024
1.785	0.20307	0.69024
6.455	0.20317	0.69024
6.925	0.20357	0.69024
8.075	0.2039	0.69024
1.455	0.20402	0.69024
2.685	0.20559	0.69258
9.615	0.20613	0.69258
6.215	0.20811	0.69268
7.495	0.2085	0.69268
9.395	0.20928	0.69268
8.225	0.20938	0.69268
1.135	0.21017	0.69268
5.695	0.21148	0.69268
5.385	0.21244	0.69268
3.885	0.21314	0.69268
9.575	0.21339	0.69268
9.355	0.21566	0.69268
2.645	0.21629	0.69268
5.125	0.2166	0.69268
6.835	0.21688	0.69268
7.715	0.2169	0.69268
6.205	0.21728	0.69268
4.695	0.21798	0.69268
6.565	0.21833	0.69268
5.645	0.219	0.69268
2.755	0.22107	0.69649
5.615	0.22381	0.69649
8.505	0.22392	0.69649
9.265	0.22398	0.69649
5.195	0.22499	0.69649
1.375	0.22561	0.69649
6.535	0.22574	0.69649
7.815	0.22595	0.69649
7.805	0.22895	0.70152

5.115	0.22902	0.70152
9.005	0.23383	0.71399
7.515	0.23538	0.71646
7.795	0.23849	0.72285
7.055	0.23896	0.72285
4.315	0.24054	0.72535
0.755	0.24144	0.72581
7.785	0.24495	0.72949
7.235	0.24511	0.72949
3.585	0.24517	0.72949
5.845	0.24567	0.72949
5.915	0.24696	0.73084
5.595	0.24763	0.73084
9.385	0.25476	0.74915
1.235	0.25591	0.74915
2.675	0.25688	0.74915
8.985	0.257	0.74915
6.185	0.25769	0.74915
2.405	0.26136	0.75569
2.185	0.26156	0.75569
8.545	0.26252	0.75569
2.285	0.26305	0.75569
7.835	0.26581	0.75961
9.545	0.26648	0.75961
9.435	0.26676	0.75961
7.445	0.27215	0.76107
0.985	0.27304	0.76107
5.455	0.27359	0.76107
5.565	0.27402	0.76107
9.515	0.27402	0.76107
2.895	0.27465	0.76107
9.245	0.27537	0.76107
6.235	0.27538	0.76107
2.355	0.27606	0.76107
5.555	0.27653	0.76107
9.165	0.27796	0.76107
6.225	0.27907	0.76107
1.015	0.27971	0.76107
8.535	0.27993	0.76107
3.935	0.2801	0.76107
1.615	0.28043	0.76107

9.585	0.2806	0.76107
5.975	0.28193	0.76181
5.775	0.28315	0.76181
8.945	0.28323	0.76181
3.865	0.28407	0.76196
7.975	0.28567	0.76375
10.275	0.28631	0.76375
5.625	0.28819	0.76498
9.235	0.28882	0.76498
7.305	0.28913	0.76498
5.985	0.29107	0.76586
6.105	0.29295	0.76586
3.945	0.29385	0.76586
6.165	0.29421	0.76586
5.305	0.29488	0.76586
4.535	0.29515	0.76586
8.935	0.29699	0.76586
8.515	0.29734	0.76586
6.195	0.2974	0.76586
7.095	0.29804	0.76586
9.805	0.29862	0.76586
10.055	0.29893	0.76586
7.755	0.30214	0.76628
5.585	0.30378	0.76628
5.445	0.30505	0.76628
6.175	0.30507	0.76628
3.415	0.30635	0.76628
5.345	0.30779	0.76628
4.275	0.30821	0.76628
8.995	0.30859	0.76628
5.375	0.30864	0.76628
1.075	0.30901	0.76628
0.665	0.30973	0.76628
10.405	0.31001	0.76628
9.645	0.31268	0.76628
5.165	0.31299	0.76628
7.605	0.31344	0.76628
6.135	0.31383	0.76628
9.225	0.3141	0.76628
4.405	0.31585	0.76628
2.535	0.31648	0.76628

2.615	0.31671	0.76628
10.345	0.31672	0.76628
1.335	0.31773	0.76628
9.535	0.31892	0.76628
5.575	0.31948	0.76628
7.965	0.32202	0.76628
9.455	0.32215	0.76628
6.305	0.32239	0.76628
7.455	0.32257	0.76628
10.205	0.32279	0.76628
2.965	0.32299	0.76628
4.515	0.32356	0.76628
2.915	0.32576	0.76791
5.055	0.32634	0.76791
6.975	0.32766	0.76791
2.745	0.3277	0.76791
2.575	0.3282	0.76791
9.035	0.33197	0.77368
9.635	0.33713	0.77368
5.635	0.33745	0.77368
9.375	0.33842	0.77368
8.855	0.33858	0.77368
1.755	0.33879	0.77368
7.985	0.33925	0.77368
1.025	0.33943	0.77368
0.725	0.33945	0.77368
9.785	0.34062	0.77368
3.765	0.34138	0.77368
0.715	0.34275	0.77368
6.965	0.34341	0.77368
2.295	0.34349	0.77368
4.045	0.34399	0.77368
10.335	0.34418	0.77368
3.245	0.34488	0.77368
2.845	0.34501	0.77368
9.935	0.34733	0.77708
4.425	0.34988	0.77932
1.125	0.35083	0.77932
10.255	0.35133	0.77932
6.755	0.35154	0.77932
6.985	0.35322	0.78092

9.685	0.35402	0.78092
8.055	0.35467	0.78092
5.045	0.35602	0.78212
2.315	0.35718	0.78248
5.955	0.35857	0.78248
7.465	0.3586	0.78248
9.465	0.36038	0.78296
4.195	0.36055	0.78296
3.175	0.36201	0.78296
7.245	0.36205	0.78296
5.895	0.36391	0.78383
9.525	0.36407	0.78383
9.735	0.36575	0.78399
9.195	0.36612	0.78399
7.045	0.36695	0.78399
5.535	0.36761	0.78399
0.745	0.36906	0.78399
6.935	0.36913	0.78399
7.535	0.36979	0.78399
3.605	0.37369	0.78904
2.725	0.37524	0.78904
1.325	0.37603	0.78904
2.485	0.37671	0.78904
5.035	0.3769	0.78904
8.205	0.37705	0.78904
2.365	0.37885	0.7911
7.405	0.38142	0.79388
3.965	0.38181	0.79388
4.355	0.3849	0.79676
8.915	0.38539	0.79676
2.015	0.38566	0.79676
10.285	0.38954	0.80188
7.995	0.38992	0.80188
6.005	0.3922	0.80188
0.675	0.39352	0.80188
0.595	0.39444	0.80188
10.415	0.3945	0.80188
9.775	0.39549	0.80188
0.925	0.39709	0.80188
8.975	0.39752	0.80188
8.275	0.39903	0.80188

8.625	0.3999	0.80188
8.725	0.40033	0.80188
3.225	0.40124	0.80188
2.905	0.40287	0.80188
3.785	0.40435	0.80188
0.655	0.40555	0.80188
7.415	0.40558	0.80188
10.395	0.4058	0.80188
1.035	0.40755	0.80188
1.995	0.40758	0.80188
0.645	0.40804	0.80188
10.425	0.40865	0.80188
7.345	0.40911	0.80188
6.125	0.40934	0.80188
7.145	0.4097	0.80188
8.035	0.41036	0.80188
8.635	0.41135	0.80188
4.945	0.41185	0.80188
2.305	0.41209	0.80188
5.715	0.41292	0.80189
6.665	0.41446	0.80267
4.035	0.41511	0.80267
5.415	0.4162	0.80267
5.515	0.41663	0.80267
1.305	0.41983	0.80423
9.795	0.42019	0.80423
9.215	0.42021	0.80423
5.465	0.42075	0.80423
7.075	0.42188	0.8048
2.425	0.42487	0.80835
0.735	0.4254	0.80835
7.725	0.42662	0.80909
9.745	0.43057	0.81498
7.905	0.43551	0.82219
4.465	0.43608	0.82219
5.945	0.43821	0.82309
1.725	0.43933	0.82309
3.365	0.44091	0.82309
9.255	0.44117	0.82309
4.995	0.44401	0.82309
8.865	0.4451	0.82309

7.825	0.44779	0.82309
8.715	0.4478	0.82309
3.995	0.44937	0.82309
7.845	0.44987	0.82309
1.835	0.45	0.82309
0.885	0.45083	0.82309
0.705	0.45089	0.82309
0.535	0.45155	0.82309
5.795	0.45238	0.82309
8.525	0.45298	0.82309
8.015	0.45367	0.82309
7.355	0.45376	0.82309
2.715	0.45381	0.82309
2.665	0.45448	0.82309
2.605	0.45752	0.82309
5.725	0.45808	0.82309
3.275	0.46078	0.82309
9.095	0.46115	0.82309
3.205	0.46176	0.82309
2.455	0.46348	0.82309
6.075	0.46389	0.82309
7.875	0.46403	0.82309
2.035	0.46571	0.82309
1.225	0.46639	0.82309
6.735	0.46825	0.82309
5.335	0.46833	0.82309
6.825	0.46937	0.82309
2.235	0.46995	0.82309
7.915	0.47115	0.82309
3.055	0.47159	0.82309
9.565	0.47261	0.82309
9.675	0.47275	0.82309
10.325	0.47368	0.82309
5.505	0.47439	0.82309
3.925	0.47507	0.82309
9.715	0.47561	0.82309
1.365	0.47678	0.82309
5.785	0.47704	0.82309
7.315	0.47755	0.82309
9.965	0.47775	0.82309
6.875	0.47821	0.82309

0.585	0.4783	0.82309
3.325	0.47848	0.82309
8.175	0.47967	0.82309
1.555	0.47978	0.82309
1.355	0.48204	0.8255
2.025	0.48378	0.82616
0.525	0.48413	0.82616
2.875	0.48835	0.82991
2.505	0.48946	0.82991
10.165	0.48959	0.82991
3.025	0.49001	0.82991
0.975	0.49092	0.82991
0.685	0.49154	0.82991
2.395	0.4923	0.82991
0.575	0.49364	0.83071
4.255	0.49524	0.83197
5.355	0.49882	0.8332
3.685	0.49885	0.8332
2.135	0.49959	0.8332
3.835	0.50101	0.8332
3.105	0.50269	0.8332
1.265	0.50357	0.8332
4.025	0.50417	0.8332
9.915	0.50569	0.8332
8.925	0.50669	0.8332
1.095	0.50685	0.8332
9.955	0.50739	0.8332
5.015	0.50746	0.8332
0.695	0.50749	0.8332
4.145	0.5081	0.8332
6.775	0.50969	0.8332
5.735	0.5097	0.8332
9.015	0.51224	0.83577
0.635	0.51358	0.83577
5.525	0.51386	0.83577
8.895	0.51644	0.83856
1.775	0.51904	0.84138
0.605	0.52151	0.84398
7.165	0.52488	0.84678
0.515	0.52608	0.84678
8.965	0.52699	0.84678

8.885	0.52781	0.84678
9.945	0.52789	0.84678
8.845	0.52847	0.84678
2.325	0.53102	0.84826
10.265	0.53117	0.84826
1.715	0.53222	0.84826
3.535	0.53401	0.84826
1.405	0.53435	0.84826
1.255	0.53538	0.84826
7.735	0.53551	0.84826
9.825	0.53842	0.84894
8.905	0.53882	0.84894
2.435	0.53892	0.84894
10.125	0.53944	0.84894
7.485	0.54256	0.85075
8.295	0.54303	0.85075
8.755	0.54322	0.85075
2.795	0.54556	0.85199
9.995	0.54576	0.85199
1.625	0.55103	0.85709
3.315	0.55104	0.85709
2.115	0.55168	0.85709
3.295	0.55326	0.85817
10.365	0.55768	0.86031
5.925	0.55846	0.86031
9.075	0.55897	0.86031
5.485	0.55897	0.86031
0.615	0.56044	0.86031
7.595	0.56045	0.86031
10.295	0.56084	0.86031
5.885	0.56405	0.86387
1.635	0.56698	0.86565
3.875	0.567	0.86565
7.105	0.56797	0.86577
6.475	0.5697	0.86655
6.055	0.57058	0.86655
2.635	0.57154	0.86655
7.945	0.57205	0.86655
2.495	0.57373	0.86775
0.545	0.57629	0.86882
0.965	0.5765	0.86882

1.915	0.57712	0.86882
4.295	0.58645	0.88037
10.485	0.58814	0.88037
3.035	0.58831	0.88037
3.185	0.58935	0.88037
3.195	0.59179	0.88037
3.955	0.59412	0.88037
9.695	0.59701	0.88037
8.805	0.59751	0.88037
4.565	0.59788	0.88037
2.075	0.59866	0.88037
0.505	0.59881	0.88037
8.955	0.59921	0.88037
2.225	0.59967	0.88037
9.205	0.60025	0.88037
8.585	0.60063	0.88037
1.005	0.60112	0.88037
1.585	0.6015	0.88037
3.095	0.60193	0.88037
5.475	0.60243	0.88037
2.975	0.60391	0.88037
1.675	0.60471	0.88037
10.445	0.60508	0.88037
1.765	0.60565	0.88037
5.855	0.60736	0.8814
6.025	0.60898	0.8814
5.255	0.61006	0.8814
4.585	0.6128	0.8814
6.295	0.61455	0.8814
9.835	0.61499	0.8814
3.335	0.61529	0.8814
1.645	0.61612	0.8814
1.315	0.61681	0.8814
10.355	0.61758	0.8814
0.945	0.61822	0.8814
5.095	0.6194	0.8814
2.335	0.62114	0.8814
3.015	0.62146	0.8814
1.295	0.6224	0.8814
6.815	0.62249	0.8814
1.465	0.623	0.8814

8.565	0.62445	0.8814
7.135	0.6246	0.8814
1.685	0.62496	0.8814
6.575	0.62542	0.8814
2.555	0.62828	0.88341
7.885	0.62894	0.88341
2.345	0.62958	0.88341
8.605	0.63075	0.88378
3.235	0.63216	0.88448
10.235	0.63799	0.88977
9.605	0.63836	0.88977
6.635	0.64092	0.88977
6.155	0.64098	0.88977
7.705	0.64287	0.88977
5.495	0.64298	0.88977
1.515	0.64358	0.88977
2.005	0.64406	0.88977
7.865	0.64442	0.88977
9.845	0.64601	0.88977
2.105	0.64602	0.88977
1.415	0.64951	0.89098
3.805	0.6499	0.89098
1.845	0.65024	0.89098
9.045	0.65057	0.89098
10.225	0.65184	0.89145
3.085	0.65348	0.89171
3.305	0.65444	0.89171
3.115	0.65478	0.89171
5.825	0.65896	0.89506
7.745	0.65908	0.89506
8.705	0.66135	0.89688
8.655	0.66276	0.89733
7.425	0.66356	0.89733
7.565	0.66461	0.89733
7.395	0.66567	0.89733
8.665	0.667	0.89733
9.125	0.6673	0.89733
1.705	0.66815	0.89733
8.685	0.67152	0.89801
4.095	0.67246	0.89801
6.015	0.67272	0.89801

0.895	0.6742	0.89801
4.495	0.677	0.89801
2.785	0.67747	0.89801
4.965	0.67747	0.89801
6.275	0.67797	0.89801
6.465	0.67828	0.89801
10.385	0.67838	0.89801
7.185	0.67893	0.89801
10.105	0.68074	0.89801
4.645	0.68158	0.89801
1.475	0.6816	0.89801
6.035	0.68336	0.89911
4.175	0.68487	0.89987
1.495	0.68816	0.90248
4.285	0.68872	0.90248
8.615	0.68964	0.90248
1.285	0.69246	0.90377
4.955	0.69328	0.90377
6.745	0.69341	0.90377
7.475	0.69878	0.90848
8.305	0.6989	0.90848
5.765	0.70032	0.90911
8.875	0.70274	0.91089
8.025	0.70357	0.91089
0.955	0.70563	0.91234
5.155	0.70816	0.91259
3.065	0.70851	0.91259
2.125	0.70951	0.91259
7.175	0.70998	0.91259
9.765	0.71052	0.91259
10.045	0.71201	0.91263
9.705	0.71508	0.91263
9.865	0.71662	0.91263
8.245	0.71816	0.91263
9.725	0.71827	0.91263
6.865	0.71838	0.91263
9.975	0.71857	0.91263
4.265	0.71884	0.91263
8.595	0.71919	0.91263
1.875	0.71995	0.91263
9.055	0.72328	0.91271

7.935	0.72399	0.91271
4.105	0.72439	0.91271
8.795	0.7246	0.91271
5.865	0.72472	0.91271
0.565	0.72597	0.91295
6.655	0.7274	0.91295
5.835	0.72786	0.91295
2.565	0.73033	0.91295
4.205	0.73053	0.91295
6.805	0.73129	0.91295
4.885	0.73237	0.91295
5.755	0.73243	0.91295
10.495	0.73495	0.91487
4.075	0.73586	0.91487
9.855	0.73687	0.91496
8.745	0.73913	0.9166
1.925	0.74297	0.92019
2.995	0.74425	0.92059
10.035	0.7472	0.92307
4.475	0.7482	0.92313
0.555	0.74946	0.92351
3.005	0.75119	0.92447
0.935	0.75253	0.92493
9.885	0.75347	0.92493
3.215	0.75489	0.9255
6.145	0.75845	0.92598
1.275	0.7587	0.92598
2.375	0.75942	0.92598
1.595	0.7607	0.92598
9.085	0.76232	0.92598
5.815	0.76294	0.92598
5.805	0.76295	0.92598
3.735	0.76373	0.92598
5.935	0.76386	0.92598
7.575	0.76772	0.92949
9.815	0.76872	0.92955
1.905	0.77228	0.9327
4.385	0.77472	0.93448
1.665	0.77774	0.93611
4.055	0.778	0.93611
8.695	0.77925	0.93644

10.155	0.78064	0.93696
1.565	0.78499	0.93918
1.865	0.78525	0.93918
10.465	0.78567	0.93918
2.415	0.78741	0.93918
4.065	0.78981	0.93918
7.895	0.79084	0.93918
1.485	0.79139	0.93918
9.905	0.79186	0.93918
1.955	0.79193	0.93918
7.155	0.79297	0.93918
3.075	0.79313	0.93918
5.745	0.79665	0.9422
4.015	0.8002	0.94254
7.855	0.80021	0.94254
8.285	0.80056	0.94254
6.615	0.80113	0.94254
1.605	0.80179	0.94254
8.005	0.80432	0.94437
1.975	0.80759	0.94682
6.725	0.80836	0.94682
1.065	0.80946	0.94698
6.675	0.81176	0.94813
1.885	0.8124	0.94813
3.705	0.81341	0.94816
2.775	0.81441	0.94819
6.605	0.81728	0.9504
6.095	0.81833	0.95048
8.785	0.82161	0.95128
9.875	0.82163	0.95128
10.005	0.82196	0.95128
3.595	0.82484	0.95295
4.125	0.82537	0.95295
8.735	0.82657	0.9532
10.245	0.8286	0.95419
9.445	0.82939	0.95419
1.445	0.83303	0.95724
3.165	0.83801	0.9611
9.655	0.83986	0.9611
7.265	0.84077	0.9611
4.985	0.84155	0.9611

2.085	0.84201	0.9611
7.955	0.84289	0.9611
4.335	0.84351	0.9611
4.365	0.84447	0.9611
3.135	0.84638	0.9611
3.855	0.84647	0.9611
2.625	0.84852	0.9611
10.085	0.85049	0.9611
2.445	0.8526	0.9611
4.115	0.85281	0.9611
6.895	0.8538	0.9611
10.145	0.85392	0.9611
5.365	0.85476	0.9611
8.065	0.85507	0.9611
6.785	0.85519	0.9611
3.125	0.85785	0.96199
6.685	0.85904	0.96199
6.945	0.86106	0.96199
7.255	0.86256	0.96199
4.375	0.86276	0.96199
6.795	0.86341	0.96199
10.475	0.86386	0.96199
2.195	0.86459	0.96199
6.045	0.8649	0.96199
3.145	0.86699	0.96322
1.345	0.86817	0.96342
1.505	0.87032	0.9647
10.095	0.8714	0.9648
2.065	0.87612	0.96892
4.895	0.88055	0.97161
6.955	0.88055	0.97161
3.795	0.88484	0.97524
5.315	0.8869	0.97626
1.895	0.88779	0.97626
1.985	0.89021	0.97667
7.115	0.89046	0.97667
5.245	0.89224	0.97667
10.065	0.89257	0.97667
4.155	0.89319	0.97667
4.595	0.89517	0.97707
1.055	0.89556	0.97707

3.695	0.89901	0.9777
6.765	0.90262	0.9777
7.555	0.90305	0.9777
7.325	0.90385	0.9777
4.505	0.90417	0.9777
2.825	0.90565	0.9777
7.525	0.90577	0.9777
9.985	0.90632	0.9777
1.965	0.90641	0.9777
6.285	0.90849	0.9777
2.735	0.90963	0.9777
1.945	0.91009	0.9777
8.255	0.91091	0.9777
6.885	0.91286	0.9777
2.855	0.91313	0.9777
5.995	0.91357	0.9777
10.115	0.91598	0.9777
5.005	0.91618	0.9777
2.045	0.91673	0.9777
7.365	0.9188	0.9777
10.135	0.91888	0.9777
0.995	0.91985	0.9777
6.625	0.92028	0.9777
6.485	0.9203	0.9777
9.145	0.92183	0.97824
10.315	0.92457	0.97994
2.985	0.92545	0.97994
6.645	0.92704	0.98056
10.455	0.92971	0.98202
6.585	0.93044	0.98202
7.545	0.93364	0.98391
4.245	0.93485	0.98391
2.585	0.93527	0.98391
6.695	0.93909	0.98601
8.765	0.94139	0.98601
4.415	0.94183	0.98601
10.305	0.94211	0.98601
5.025	0.94311	0.98601
9.065	0.94363	0.98601
1.045	0.94438	0.98601
4.575	0.94674	0.98742

5.175	0.94788	0.98754
7.505	0.95	0.98864
8.575	0.95211	0.98864
5.905	0.95223	0.98864
2.835	0.95401	0.98864
10.015	0.95402	0.98864
1.575	0.95581	0.98944
8.645	0.96013	0.99099
10.375	0.96142	0.99099
7.295	0.96247	0.99099
6.595	0.96285	0.99099
0.625	0.96289	0.99099
7.925	0.96344	0.99099
2.595	0.96809	0.99303
8.675	0.96906	0.99303
4.485	0.97066	0.99303
7.435	0.97071	0.99303
9.155	0.97137	0.99303
10.025	0.97155	0.99303
10.075	0.97494	0.99464
2.055	0.97518	0.99464
1.695	0.97734	0.9958
9.895	0.97894	0.99599
2.385	0.98098	0.99599
1.935	0.98119	0.99599
3.155	0.98163	0.99599
8.775	0.98338	0.99613
7.585	0.98449	0.99613
4.325	0.98485	0.99613
9.755	0.98591	0.99616
4.975	0.98975	0.99735
4.005	0.99098	0.99735
1.855	0.99255	0.99735
3.285	0.99282	0.99735
1.655	0.99322	0.99735
4.165	0.99419	0.99735
4.395	0.99593	0.99735
7.125	0.99637	0.99735
6.115	0.99693	0.99735
10.435	0.99735	0.99735

Table S4. Bins with a VIP value >1.

Primary ID	VIP value
**3.7587**	6.72868
**3.7787**	6.5927
**3.7487**	5.47052
**3.3978**	4.77776
**3.8689**	4.69003
**3.5582**	4.66386
**3.879**	4.63232
**3.4279**	4.56277
**3.5782**	4.5433
**3.8288**	4.43507
**3.4078**	4.42954
**3.5482**	4.35305
**3.7186**	4.34885
**3.9792**	4.31232
**3.5381**	4.18791
**3.9692**	4.15859
**3.7386**	4.03898
**3.7687**	4.01822
**3.8088**	3.96129
**3.7286**	3.83478
**3.8589**	3.6794
**3.8188**	3.53439
**3.7988**	3.47661
**3.3878**	3.47426
**3.7888**	3.31498
**3.909**	3.25652
**3.9592**	3.22423
**3.2174**	3.01484
**3.2575**	2.9245
**3.4379**	2.91914
**3.9491**	2.90698
**3.4178**	2.89646
**4.0293**	2.86184
**3.5281**	2.84587
**5.3626**	2.81285
**3.9892**	2.77331
**3.8489**	2.67889
**3.889**	2.61609

**5.3726**	2.59426
**3.9391**	2.58023
**3.5181**	2.55566
**3.9993**	2.54803
**3.6985**	2.478
**2.3653**	2.45845
**3.4479**	2.44792
**3.2073**	2.44338
**3.2474**	2.37422
**5.2122**	2.25688
**4.6308**	2.22502
**3.5081**	2.21836
**3.8389**	2.18353
**3.4579**	2.11282
**2.3553**	2.10415
**0.89172**	2.09726
**3.9291**	2.08958
**4.0193**	2.06032
**4.9215**	1.99898
**5.3525**	1.99549
**4.6408**	1.97572
**1.3428**	1.97494
**3.3778**	1.90278
**3.2675**	1.8495
**3.6785**	1.84152
**3.7086**	1.81418
**4.0093**	1.77359
**3.2374**	1.75409
**3.1873**	1.69789
**3.478**	1.69375
**5.3425**	1.68348
**4.0594**	1.66406
**3.5682**	1.66043
**4.1897**	1.63919
**4.1196**	1.63212
**5.2323**	1.56819
**3.468**	1.56518
**5.3826**	1.56367
**2.5858**	1.55073
**3.1672**	1.53326
**2.4054**	1.47601

**2.3452**	1.43307
**3.9191**	1.4188
**4.5506**	1.40794
**2.3853**	1.3788
**5.2924**	1.37321
**1.9844**	1.37158
**3.3377**	1.35589
**4.9415**	1.35498
**3.488**	1.35115
**3.5883**	1.31293
**5.3325**	1.30411
**5.3926**	1.29537
**3.2274**	1.29124
**5.2423**	1.28852
**5.3225**	1.28285
**1.022**	1.28135
**5.1821**	1.27627
**1.4431**	1.25553
**5.2222**	1.24237
**4.2198**	1.22409
**0.97191**	1.20781
**4.0995**	1.20484
**1.3328**	1.16618
**5.4227**	1.15877
**3.1973**	1.15135
**7.3674**	1.13253
**7.3775**	1.12572
**4.2298**	1.12107
**3.3076**	1.11992
**4.1396**	1.08727
**5.3024**	1.08489
**3.3677**	1.08039
**4.0895**	1.07656
**5.2824**	1.06644
**2.3252**	1.05664
**1.012**	1.04539
**2.3152**	1.02826
**1.0321**	1.01535
**0.87167**	1.00407
**1.0421**	0.985516
**0.86165**	0.984678

**3.498**	0.983446
**3.3276**	0.975417
**5.0919**	0.964186
**6.8562**	0.961128
**0.85162**	0.957731
**4.3501**	0.955035
**2.0545**	0.953991
**2.3954**	0.952208
**1.4531**	0.950761
**4.1496**	0.949782
**6.8762**	0.942047
**5.1721**	0.940816
**2.3352**	0.938743
**4.1697**	0.934775
**7.999**	0.934391
**4.0494**	0.933145
**5.1922**	0.927758
**4.6208**	0.921142
**2.3753**	0.910009
**4.4504**	0.907766
**4.9516**	0.9048
**0.88169**	0.898205
**3.3176**	0.897502
**5.3124**	0.89304
**4.0795**	0.89175
**2.255**	0.889562
**1.5233**	0.881272
**6.345**	0.87986
**4.6508**	0.878094
**3.2976**	0.876427
**1.9743**	0.874314
**4.6609**	0.873594
**4.1797**	0.866361
**4.1095**	0.862267
**0.96189**	0.859091
**0.93182**	0.851376
**1.3528**	0.8507
**3.2875**	0.841593
**4.0394**	0.841325
**3.6885**	0.839106
**0.94184**	0.831618

**3.899**	0.814262
**3.0069**	0.812953
**5.2724**	0.801985
**0.95186**	0.797997
**0.90174**	0.794848
**3.0269**	0.793517
**4.9115**	0.79288
**7.3875**	0.789991
**2.0445**	0.787228
**2.4856**	0.782063
**2.4756**	0.780758
**7.9889**	0.778408
**1.433**	0.77606
**1.9142**	0.774957
**4.9315**	0.772925
**4.5606**	0.764048
**1.4631**	0.758725
**3.0169**	0.756235
**2.3051**	0.75512
**2.9267**	0.754509
**4.1296**	0.73478
**4.0694**	0.732621
**2.5758**	0.722211
**3.2775**	0.720882
**1.7438**	0.718192
**1.9944**	0.717745
**5.4327**	0.713785
**2.9968**	0.71184
**6.8963**	0.710771
**4.32**	0.710337
**1.1123**	0.704125
**5.4027**	0.699427
**2.0345**	0.695309
**3.3577**	0.685034
**5.2022**	0.68322
**6.8863**	0.679838
**2.2651**	0.675829
**4.4403**	0.675006
**1.9643**	0.673238
**3.1773**	0.672374
**0.8416**	0.66599

**4.3802**	0.650384
**1.7338**	0.645238
**2.656**	0.642226
**2.0245**	0.64054
**4.29**	0.637454
**1.7037**	0.636273
**7.0467**	0.616571
**1.8741**	0.616407
**3.3477**	0.609589
**1.8841**	0.608192
**5.4528**	0.606229
**2.1347**	0.604765
**0.92179**	0.603805
**6.8662**	0.603082
**3.1271**	0.600787
**3.0369**	0.598397
**4.2098**	0.595803
**2.5959**	0.592509
**1.8942**	0.589647
**2.6861**	0.587937
**2.6961**	0.585843
**4.3702**	0.583925
**0.99196**	0.58352
**8.3398**	0.58339
**2.0746**	0.577385
**2.7262**	0.57589
**3.087**	0.573491
**1.0822**	0.573467
**4.1997**	0.570273
**2.1247**	0.570196
**2.4956**	0.568262
**1.403**	0.567525
**3.1171**	0.564721
**0.98194**	0.563535
**2.2951**	0.563492
**0.91177**	0.562184
**2.4254**	0.560838
**1.2426**	0.559876
**1.814**	0.559296
**1.5333**	0.558082
**5.2523**	0.555539

**1.413**	0.553555
**2.1047**	0.551721
**4.1597**	0.550673
**3.0971**	0.550373
**1.9042**	0.550031
**2.0144**	0.54846
**1.6937**	0.547747
**4.3**	0.54293
**2.245**	0.540498
**2.4655**	0.538974
**5.1621**	0.538703
**2.9868**	0.532548
**4.4103**	0.52822
**2.4355**	0.527472
**6.9063**	0.520986
**2.2851**	0.520523
**4.5005**	0.519808
**7.5078**	0.518579
**5.2623**	0.51845
**5.4628**	0.518152
**4.4905**	0.517832
**4.6809**	0.516427
**7.3073**	0.515355
**3.0469**	0.515348
**2.1448**	0.515216
**1.5132**	0.511902
**7.5178**	0.511777
**1.2225**	0.510469
**2.0846**	0.508569
**2.9467**	0.507923
**5.4428**	0.507577
**2.7663**	0.50742
**1.6135**	0.505344
**2.1147**	0.505129
**2.2751**	0.502942
**2.2149**	0.502917
**3.057**	0.501594
**2.0946**	0.500845
**5.4127**	0.497389
**7.6381**	0.493881
**4.2699**	0.493534

**1.4731**	0.492558
**1.9543**	0.489368
**4.4604**	0.488719
**1.7137**	0.488638
**7.3975**	0.488007
**3.1071**	0.487383
**1.5032**	0.487021
**2.8665**	0.486839
**1.8039**	0.486608
**2.5658**	0.486454
**4.4704**	0.485714
**7.4075**	0.485189
**1.5734**	0.48327
**5.1521**	0.480834
**4.5406**	0.480372
**4.6709**	0.479968
**2.7562**	0.473978
**2.7763**	0.472775
**4.9616**	0.472704
**6.9163**	0.472192
**1.1223**	0.470539
**4.6909**	0.470153
**7.2973**	0.466781
**2.9567**	0.46605
**7.3474**	0.466026
**2.5056**	0.463543
**6.365**	0.463294
**4.8914**	0.463106
**7.1669**	0.462016
**2.7963**	0.461688
**1.5433**	0.460272
**1.8641**	0.456061
**1.6536**	0.452651
**1.6435**	0.452249
**1.6836**	0.451477
**3.1472**	0.450672
**5.0217**	0.450556
**1.2325**	0.450291
**2.9367**	0.448541
**3.1572**	0.448401
**1.3027**	0.448338

**1.7538**	0.447875
**1.9443**	0.447274
**2.9768**	0.446825
**4.5706**	0.446547
**5.0719**	0.443749
**1.6736**	0.4407
**2.5157**	0.440317
**2.0044**	0.439088
**5.4728**	0.434152
**1.824**	0.432806
**4.2499**	0.431756
**1.2125**	0.427699
**7.0366**	0.427529
**2.0646**	0.42645
**2.4555**	0.426357
**2.7863**	0.425201
**5.142**	0.425059
**2.5558**	0.424839
**2.8866**	0.423532
**5.533**	0.423306
**5.0618**	0.423025
**1.6335**	0.422203
**0.83157**	0.420567
**1.2526**	0.418967
**1.7237**	0.418394
**3.077**	0.41809
**4.3601**	0.416245
**4.5105**	0.416006
**1.9342**	0.41353
**1.6636**	0.406059
**4.3301**	0.405491
**7.9789**	0.40509
**2.8164**	0.404736
**2.4154**	0.403653
**1.5633**	0.403019
**1.0521**	0.402681
**7.6281**	0.400256
**7.177**	0.39988
**1.3127**	0.399635
**1.002**	0.399443
**4.2398**	0.39857

**4.2599**	0.398357
**4.9015**	0.396757
**2.4455**	0.394629
**5.4829**	0.392604
**3.1372**	0.391337
**1.4932**	0.389881
**7.5379**	0.386777
**4.3401**	0.385988
**7.8987**	0.384953
**2.8765**	0.384247
**5.0819**	0.383544
**4.6107**	0.382359
**2.5457**	0.380461
**2.6059**	0.379584
**1.6235**	0.378702
**7.2772**	0.378608
**2.676**	0.377421
**8.2696**	0.376372
**7.0567**	0.375542
**4.6007**	0.37457
**4.5807**	0.373171
**2.646**	0.372277
**2.1548**	0.372099
**1.2626**	0.371147
**2.666**	0.370549
**2.9668**	0.369314
**1.5934**	0.366634
**1.1022**	0.365036
**1.2927**	0.364928
**8.2496**	0.363838
**7.3574**	0.362901
**4.4303**	0.361852
**2.8565**	0.360198
**1.3929**	0.360098
**2.7462**	0.358546
**5.6633**	0.356925
**7.1569**	0.356229
**1.0922**	0.355245
**8.3298**	0.353858
**4.4804**	0.352821
**7.2672**	0.350078

**2.8465**	0.349335
**1.2827**	0.348919
**1.6034**	0.347542
**6.8462**	0.346091
**7.7083**	0.345128
**1.3829**	0.343414
**1.0621**	0.343167
**1.9242**	0.342289
**2.8064**	0.341062
**0.82155**	0.339847
**1.7939**	0.339456
**1.423**	0.335926
**7.618**	0.335046
**8.3498**	0.333957
**3.067**	0.333805
**2.2049**	0.333666
**6.0643**	0.331658
**2.7061**	0.329831
**4.4002**	0.329519
**7.6782**	0.329039
**7.7183**	0.328038
**4.31**	0.327287
**5.6733**	0.326011
**1.7638**	0.325617
**4.9917**	0.325612
**6.0743**	0.32557
**2.8364**	0.325077
**1.844**	0.324828
**7.4978**	0.323247
**7.9088**	0.322297
**7.6882**	0.321954
**6.355**	0.318471
**2.7362**	0.318209
**5.4929**	0.317878
**0.81152**	0.317704
**6.3951**	0.317612
**1.8541**	0.316236
**2.8264**	0.31623
**8.3899**	0.315765
**6.5053**	0.315671
**2.235**	0.31474

**6.8261**	0.312959
**1.3629**	0.312426
**8.3197**	0.312394
**2.9166**	0.312175
**1.0722**	0.311737
**5.8738**	0.310742
**7.5278**	0.310623
**2.225**	0.310077
**2.6159**	0.309068
**4.3902**	0.306942
**1.834**	0.306797
**8.2195**	0.306559
**7.2872**	0.305678
**8.3999**	0.305391
**1.5834**	0.305355
**4.5205**	0.305212
**6.2648**	0.30259
**8.3598**	0.301825
**7.187**	0.301305
**5.7836**	0.300613
**2.5357**	0.30025
**5.5129**	0.298709
**6.8161**	0.298341
**4.4203**	0.295452
**2.5257**	0.295052
**1.2726**	0.293916
**5.122**	0.293051
**1.3228**	0.291373
**7.6481**	0.291224
**2.7161**	0.289696
**5.5029**	0.288853
**2.9066**	0.28809
**8.3097**	0.287768
**5.6032**	0.286766
**2.1748**	0.283592
**7.2572**	0.283252
**6.9264**	0.282372
**6.5655**	0.281765
**6.796**	0.280638
**8.6105**	0.279523
**5.543**	0.279137

**1.4832**	0.278869
**5.553**	0.278761
**1.5533**	0.278175
**1.7739**	0.274193
**8.009**	0.273545
**6.786**	0.273497
**7.3374**	0.272168
**6.0843**	0.271003
**1.7839**	0.270944
**5.5931**	0.270575
**6.8361**	0.270026
**4.5907**	0.269739
**2.636**	0.26859
**7.6982**	0.267639
**6.4352**	0.267639
**8.2797**	0.266291
**5.523**	0.264868
**5.1019**	0.264815
**8.2596**	0.264278
**7.6581**	0.261579
**5.5631**	0.261152
**5.6433**	0.259259
**5.5831**	0.258856
**7.2471**	0.25659
**6.9364**	0.256381
**6.8061**	0.255446
**4.5306**	0.250933
**5.6533**	0.25087
**6.375**	0.2508
**5.5731**	0.249475
**5.0117**	0.247969
**5.6132**	0.247834
**6.9464**	0.247659
**7.3273**	0.247631
**5.8838**	0.246941
**2.1648**	0.246638
**7.6682**	0.24515
**6.5555**	0.243816
**1.3729**	0.243809
**7.0266**	0.241373
**4.9816**	0.24075

**8.1894**	0.240722
**5.6833**	0.240072
**7.4176**	0.239965
**7.2171**	0.239293
**5.6332**	0.239266
**5.6934**	0.238755
**8.43**	0.238455
**6.776**	0.238062
**7.3173**	0.238043
**4.2799**	0.237991
**5.0017**	0.237023
**5.0418**	0.236695
**5.6232**	0.2347
**8.6205**	0.233933
**8.1193**	0.233327
**5.7034**	0.23291
**2.6259**	0.231663
**8.3799**	0.229701
**2.1949**	0.229436
**7.1469**	0.229413
**2.8966**	0.227549
**5.0318**	0.226593
**7.2271**	0.224958
**7.9589**	0.224897
**5.0518**	0.224723
**2.1849**	0.223936
**8.0992**	0.223883
**7.608**	0.223694
**8.3699**	0.221316
**7.0667**	0.220849
**5.112**	0.220359
**7.0968**	0.219103
**8.1995**	0.21816
**7.2371**	0.217933
**4.9716**	0.215747
**8.0691**	0.214553
**8.1493**	0.213202
**7.8586**	0.212753
**7.8687**	0.212357
**7.0166**	0.212092
**8.0391**	0.211751

**8.41**	0.208737
**0.79147**	0.208333
**6.4652**	0.207084
**0.8015**	0.206599
**6.756**	0.206217
**0.78145**	0.205971
**6.0943**	0.205939
**7.7784**	0.205857
**6.766**	0.205129
**8.1594**	0.204253
**8.1393**	0.204162
**7.8787**	0.204042
**6.2848**	0.202153
**8.2295**	0.198486
**8.2997**	0.1984
**7.5679**	0.198197
**7.0767**	0.197845
**5.954**	0.197808
**7.5779**	0.197746
**8.5503**	0.1974
**0.77143**	0.196675
**5.8939**	0.196329
**7.8887**	0.196181
**8.1794**	0.196114
**7.1068**	0.195146
**8.5804**	0.194945
**8.44**	0.193295
**7.207**	0.193208
**7.588**	0.19313
**6.7459**	0.192724
**7.5579**	0.192204
**7.1269**	0.191979
**5.132**	0.191804
**8.1293**	0.191453
**7.197**	0.19067
**7.598**	0.190398
**7.9689**	0.190186
**5.7134**	0.189946
**8.2095**	0.189011
**6.2547**	0.188913
**7.5479**	0.188861

**8.0591**	0.188425
**7.9288**	0.187019
**8.019**	0.186471
**7.0868**	0.18624
**6.5855**	0.185481
**7.1168**	0.184584
**6.9564**	0.184341
**8.1694**	0.184062
**8.0892**	0.183875
**5.964**	0.183467
**8.1092**	0.18301
**6.7359**	0.181658
**7.8486**	0.181284
**8.029**	0.181011
**7.0066**	0.180679
**8.8109**	0.180623
**8.42**	0.180363
**8.0491**	0.180103
**6.4552**	0.179892
**8.5904**	0.179479
**5.7936**	0.178854
**5.9039**	0.176892
**8.821**	0.176591
**8.0792**	0.176317
**6.4051**	0.17603
**7.7283**	0.175501
**7.4577**	0.175246
**7.1369**	0.175063
**9.673**	0.174634
**6.4151**	0.174411
**6.9965**	0.17347
**6.5154**	0.172958
**7.4877**	0.172836
**0.7614**	0.172356
**7.4677**	0.172084
**9.1016**	0.17184
**7.9188**	0.170608
**6.4251**	0.170086
**8.5403**	0.169962
**6.9665**	0.169651
**6.5454**	0.167585

**5.9139**	0.16732
**7.4476**	0.167253
**9.1117**	0.166957
**6.6958**	0.166224
**8.4601**	0.166103
**6.6758**	0.165963
**6.3349**	0.165713
**6.5254**	0.164488
**6.9865**	0.164367
**6.1344**	0.164367
**8.4701**	0.163392
**5.9841**	0.163251
**8.4501**	0.163208
**7.7684**	0.162957
**6.9765**	0.162868
**6.5755**	0.162842
**8.2897**	0.162583
**10.204**	0.160047
**7.8386**	0.159642
**6.2748**	0.159469
**6.7159**	0.159456
**6.1244**	0.158943
**6.0041**	0.158706
**8.2396**	0.156154
**6.6657**	0.155326
**0.75138**	0.154404
**6.0542**	0.154147
**6.7259**	0.153915
**7.4777**	0.153831
**7.4376**	0.152485
**6.6457**	0.15178
**5.7234**	0.151224
**7.7985**	0.150452
**6.6858**	0.148696
**7.7383**	0.147464
**8.4801**	0.147419
**6.5354**	0.14543
**8.5704**	0.145398
**8.5603**	0.145144
**7.7885**	0.144995
**6.6357**	0.144768

**0.74135**	0.144614
**8.831**	0.143909
**0.73133**	0.1423
**7.7484**	0.141278
**5.9239**	0.141091
**9.3021**	0.140737
**6.1144**	0.139801
**9.3523**	0.139034
**7.7584**	0.138651
**7.9488**	0.138528
**5.7736**	0.137721
**6.7058**	0.137348
**6.6557**	0.137008
**8.6305**	0.136534
**0.7213**	0.136508
**9.0215**	0.136435
**9.3422**	0.136215
**7.8286**	0.13499
**8.4902**	0.134986
**8.5002**	0.133292
**6.2347**	0.133282
**5.944**	0.133135
**6.4853**	0.132976
**7.4276**	0.130394
**6.3149**	0.129779
**6.3249**	0.129614
**8.5303**	0.128644
**5.7335**	0.128511
**8.5102**	0.128074
**7.9388**	0.127502
**6.1445**	0.126456
**0.71128**	0.126293
**8.5202**	0.125275
**6.1946**	0.124902
**10.214**	0.124737
**6.6256**	0.124661
**6.4452**	0.124457
**9.1217**	0.123878
**6.4953**	0.123352
**0.70126**	0.122959
**6.3851**	0.122483

**0.62106**	0.122249
**0.69123**	0.12194
**6.2046**	0.121831
**6.2948**	0.121087
**5.7535**	0.120016
**6.2247**	0.119946
**6.5956**	0.119325
**5.9741**	0.119319
**6.6156**	0.118887
**6.0242**	0.118427
**0.68121**	0.118276
**0.67118**	0.11755
**5.7435**	0.117444
**7.8085**	0.117382
**0.66116**	0.116397
**8.6004**	0.116165
**0.65113**	0.11551
**0.63109**	0.115396
**0.61104**	0.115222
**6.2447**	0.114349
**0.64111**	0.11415
**9.4425**	0.113005
**6.1745**	0.111692
**6.1846**	0.111366
**9.2821**	0.111355
**0.60101**	0.110679
**8.7107**	0.110217
**9.2921**	0.109845
**0.57094**	0.107029
**0.59099**	0.106839
**7.8185**	0.105752
**0.58096**	0.105561
**6.1044**	0.105371
**6.2146**	0.105107
**6.6056**	0.105034
**0.54087**	0.10478
**0.56092**	0.104128
**0.53084**	0.104033
**0.55089**	0.103295
**9.4826**	0.102808
**0.51079**	0.102551

**0.50285**	0.102532
**0.52082**	0.10169
**9.0315**	0.100206
**8.7207**	0.098394
**6.1645**	0.097113
**6.4753**	0.095208
**5.934**	0.092209
**8.841**	0.090667
**5.8237**	0.090034
**5.8638**	0.089386
**6.3049**	0.088821
**5.8137**	0.088623
**5.8036**	0.087693
**5.8337**	0.086164
**10.194**	0.086056
**8.6706**	0.084589
**5.9941**	0.083182
**8.6606**	0.080764
**6.0142**	0.080206
**5.7635**	0.079292
**9.3122**	0.078764
**6.1545**	0.078489
**8.6806**	0.078039
**8.7307**	0.078005
**8.7007**	0.077414
**10.224**	0.076783
**9.4725**	0.075279
**8.851**	0.075125
**9.4024**	0.07394
**9.3322**	0.073462
**6.0442**	0.073202
**9.663**	0.073061
**8.6906**	0.072919
**9.4926**	0.07252
**9.0916**	0.072516
**8.8009**	0.072326
**9.3924**	0.07223
**9.4124**	0.072117
**9.3623**	0.071691
**8.7608**	0.071472
**8.9012**	0.070184

**8.9312**	0.069666
**6.0342**	0.068701
**8.8611**	0.068657
**8.9914**	0.06702
**8.9413**	0.06685
**9.2721**	0.066739
**8.9713**	0.066535
**9.653**	0.066514
**9.1718**	0.066278
**8.7408**	0.06603
**8.6405**	0.065851
**9.0415**	0.064854
**5.8437**	0.064631
**8.8911**	0.064611
**9.0014**	0.064245
**9.1818**	0.063999
**9.5026**	0.0639
**8.9513**	0.063313
**8.9212**	0.062701
**10.184**	0.062547
**9.4525**	0.061779
**9.3222**	0.061529
**8.7508**	0.061159
**9.1417**	0.060901
**10.134**	0.060875
**8.9814**	0.060733
**9.1317**	0.0605
**10.174**	0.060437
**8.7708**	0.060369
**10.164**	0.060336
**9.3723**	0.060128
**8.6506**	0.05987
**8.8711**	0.059787
**8.7909**	0.059719
**8.9112**	0.05964
**9.0515**	0.059134
**8.8811**	0.058352
**8.7809**	0.057145
**9.5928**	0.056457
**9.232**	0.056051
**9.0114**	0.055926

**10.144**	0.055905
**9.4224**	0.055807
**9.0816**	0.055435
**9.3823**	0.055125
**8.9613**	0.05448
**9.2119**	0.052973
**10.234**	0.052649
**9.242**	0.052578
**9.5327**	0.052412
**10.154**	0.052402
**9.4324**	0.052299
**9.5126**	0.052164
**9.5527**	0.052121
**9.2019**	0.052053
**10.054**	0.051852
**9.1618**	0.051776
**9.1919**	0.050822
**10.044**	0.050762
**9.5427**	0.049931
**9.252**	0.049925
**10.114**	0.049783
**9.2219**	0.049423
**9.262**	0.04931
**9.6329**	0.04886
**9.6129**	0.048757
**9.6029**	0.048397
**5.8538**	0.048283
**10.124**	0.04828
**10.064**	0.048252
**9.0615**	0.047963
**10.104**	0.047149
**9.4625**	0.047053
**9.7332**	0.046875
**9.1518**	0.046809
**9.5628**	0.046327
**10.094**	0.04627
**9.6831**	0.045831
**9.0716**	0.044539
**9.5728**	0.044154
**9.643**	0.044041
**9.5828**	0.043903

**9.9136**	0.043889
**9.8134**	0.043841
**10.285**	0.043744
**9.8033**	0.043151
**10.034**	0.043097
**9.9437**	0.043095
**9.9537**	0.042803
**9.7633**	0.041802
**9.9337**	0.041664
**9.6931**	0.041572
**10.084**	0.041217
**9.7131**	0.041204
**9.6229**	0.041043
**9.7432**	0.040786
**9.7031**	0.04076
**9.8936**	0.040593
**9.5227**	0.039728
**10.024**	0.039677
**9.8334**	0.039542
**9.7833**	0.039493
**10.345**	0.039292
**9.9036**	0.039236
**10.264**	0.038934
**9.8434**	0.038716
**9.8835**	0.03854
**10.274**	0.038467
**10.074**	0.038413
**9.7232**	0.038384
**9.9236**	0.038287
**9.8635**	0.038122
**9.7933**	0.03811
**9.9838**	0.037774
**10.415**	0.037768
**9.8735**	0.0376
**9.7532**	0.037497
**9.9637**	0.037473
**10.335**	0.037436
**10.385**	0.037141
**9.8535**	0.037072
**10.254**	0.036648
**9.9738**	0.035542

**9.7733**	0.03517
**9.8234**	0.034313
**10.425**	0.034296
**10.405**	0.033823
**10.295**	0.032761
**10.395**	0.03246
**9.9938**	0.032306
**10.315**	0.031847
**10.305**	0.03167
**10.475**	0.031083
**10.244**	0.029991
**10.014**	0.029966
**10.435**	0.028286
**10.465**	0.027414
**10.355**	0.027174
**10.495**	0.026866
**10.004**	0.026584
**10.455**	0.026561
**10.365**	0.02557
**10.445**	0.025363
**10.325**	0.025321
**10.375**	0.02437
**10.485**	0.022393

Table S5. Confusion matrix obtained from random forest using ICOSHIFT aligned data with a bin size of 0.01 ppm.

	**Ale**	**Lager**	**class.error**
Ale	16	4	0.2
Lager	4	15	0.211
